# Molecular and biochemical investigations of the anti-fatigue effects of tea polyphenols and fruit extracts of *Lycium ruthenicum* Murr. on mice with exercise-induced fatigue

**DOI:** 10.3389/fmolb.2023.1223411

**Published:** 2023-06-20

**Authors:** Yingxin Bi, Xianjun Liu, Yue Liu, Mengyuan Wang, Yaming Shan, Yuhe Yin, Xianglong Meng, Fengjie Sun, Hao Li, Zhandong Li

**Affiliations:** ^1^ College of Biological and Food Engineering, Jilin Engineering Normal University, Changchun, China; ^2^ School of Chemistry and Life Science, Changchun University of Technology, Changchun, China; ^3^ National Engineering Laboratory for AIDS Vaccine, School of Life Sciences, Jilin University, Changchun, China; ^4^ Key Laboratory for Molecular Enzymology and Engineering, The Ministry of Education, School of Life Sciences, Jilin University, Changchun, China; ^5^ Department of Burns Surgery, The First Hospital of Jilin University, Changchun, China; ^6^ School of Science and Technology, Georgia Gwinnett College, Lawrenceville, GA, United States

**Keywords:** exercise-induced, fatigue, tea polyphenols, *Lycium ruthenicum*, microRNA, lactate dehydrogenase, superoxide dismutase, tumor necrosis factor-α

## Abstract

**Background:** The molecular mechanisms regulating the therapeutic effects of plant-based ingredients on the exercise-induced fatigue (EIF) remain unclear. The therapeutic effects of both tea polyphenols (TP) and fruit extracts of *Lycium ruthenicum* (LR) on mouse model of EIF were investigated.

**Methods:** The variations in the fatigue-related biochemical factors, i.e., lactate dehydrogenase (LDH), superoxide dismutase (SOD), tumor necrosis factor-α (TNF-α), interleukin-1β (IL-1β), interleukin-2 (IL-2), and interleukin-6 (IL-6), in mouse models of EIF treated with TP and LR were determined. The microRNAs involved in the therapeutic effects of TP and LR on the treatment of mice with EIF were identified using the next-generation sequencing technology.

**Results:** Our results revealed that both TP and LR showed evident anti-inflammatory effect and reduced oxidative stress. In comparison with the control groups, the contents of LDH, TNF-α, IL-6, IL-1β, and IL-2 were significantly decreased and the contents of SOD were significantly increased in the experimental groups treated with either TP or LR. A total of 23 microRNAs (21 upregulated and 2 downregulated) identified for the first time by the high-throughput RNA sequencing were involved in the molecular response to EIF in mice treated with TP and LR. The regulatory functions of these microRNAs in the pathogenesis of EIF in mice were further explored based on Gene Ontology (GO) annotation and Kyoto Encyclopedia of Genes and Genomes (KEGG) enrichment analyses with a total of over 20,000–30,000 target genes annotated and 44 metabolic pathways enriched in the experimental groups based on GO and KEGG databases, respectively.

**Conclusion:** Our study revealed the therapeutic effects of TP and LR and identified the microRNAs involved in the molecular mechanisms regulating the EIF in mice, providing strong experimental evidence to support further agricultural development of LR as well as the investigations and applications of TP and LR in the treatment of EIF in humans, including the professional athletes.

## 1 Introduction

Fatigue is generally described as either tiredness or lack of energy, while the exercise-induced fatigue (EIF) is a normal physical reaction generally caused by either lack of exercise, prolonged exercise, unhealthy diet, or biorhythm disorders (Enoka and Duchateau, 2016; Penner and Paul, 2017), and biochemically contributed and marked by oxidative stress (Fukuda et al., 2016; Lee et al., 2018). Studies have shown that the occurrence of fatigue is caused by the complex interactions among various internal and external factors (Powers and Jackson, 2008; Reid, 2008; Finsterer and Mahjoub, 2014; Fukuda et al., 2016; Katz, 2017; Penner and Paul, 2017; Lee et al., 2018; Bagni et al., 2019; Ruiz-Schutz et al., 2019; Higgins et al., 2020), while several popular theories have been proposed to explain the molecular mechanisms underlying the pathogenesis of EIF (Tang, 1997; Xu et al., 2014; Coutinho de Oliveira et al., 2016; Cheng et al., 2020; Zhou et al., 2021). Generally, in a prolonged exercise, the energy material is not replenished in time and the physiological situations of the body is weakened, ultimately decreasing the exercise ability and eventually causing the extended muscle pain and muscle fatigue. According to the free radical theory (Cheng et al., 2015; Persson et al., 2019), the amounts of free radicals are increased in the body after the exercise, while the content of superoxide dismutase (SOD) is decreased and the content of malondialdehyde (MDA) is increased, causing the impaired physiological functions in cells and obstructed functions of tissues and organs, ultimately leading to fatigue.

Although in most cases, the fatigue could be completely relieved by taking appropriate rest or adjustment of some specific lifestyles, it is occasionally a challenge to find an appropriate way to alleviate the fatigue, in particular, under the continuous pressure of living in the current rapidly developing societies worldwide. In the traditional Chinese medicine (TCM), both acupuncture and moxibustion are applied to effectively treat various medical disorders, including fatigue (Wang et al., 2017; Wang H. et al., 2018). Furthermore, medicines based on extracts and active ingredients of traditional Chinese medicinal plants are easily accessible and suitable for large-scale, effective, and rapid remedy for fatigue worldwide (Liu et al., 2022). To date, two types of effective ingredients of Chinese medicinal plants have been widely used to treat fatigue, i.e., tea polyphenols (Liu et al., 2017) and fruit extracts of *Lycium ruthenicum* Murr. (Liu et al., 2013), suggesting promising prospects with strong clinical evidence to support the therapeutic treatments of EIF. As the main pharmacologically active ingredients in tea, the tea polyphenols account for about 30% of the dry weight (Brown, 1999; Khan and Mukhtar, 2018) and are readily available and easily absorbed by the body, with the functions of anti-oxidation, antiviral, anti-tumor, weight loss, scavenging oxygen free radicals, anti-fatigue, and regulating human metabolic activities (Niedzwiecki et al., 2016; Rothenberg et al., 2018; Lucarini et al., 2020; Mhatre et al., 2021; Ohishi et al., 2021). As a native species in the Qinghai-Tibet Plateau, plants of *L. ruthenicum* Murr. are rich in flavonoids (e.g., anthocyanins), polysaccharides, phenolic acids, carotenoids, alkaloids, and essential oils (Girard et al., 2011; Wang H. et al., 2018). Similar to tea polyphenols, fruits of *L. ruthenicum* show high medicinal significance, i.e., scavenging free radicals and anti-cancer effects (Zhang et al., 2019; Lu et al., 2020; Luo et al., 2021) as well as the treatments of high blood pressure, menstrual disorders, vision, heart diseases, and many other types of medical disorders due to its antioxidant activity (Zheng et al., 2011; Lin et al., 2015; Wang et al., 2018; Deng et al., 2020; Luan et al., 2021; Tian et al., 2021). Although the *L. ruthenicum* has shown a wide range of medicinal functions, the molecular mechanisms regulating these therapeutic effects remain unclear.

Recently, various types of microRNAs (miRNAs) have been revealed with differential expressions in many human diseases and commonly used as biomarkers and therapeutic targets to predict and treat the development of these diseases (Santulli, 2016; O’Brien et al., 2018). For example, the patients of chronic fatigue syndrome (CFS) have shown significant variations in the expression of genes encoding miRNAs that regulate cytotoxicity, cytokine secretion, and apoptosis, closely related to the high expression of immunoinflammatory genes encoding tumor necrosis factor-α (TNF-α), interleukin-6 (IL-6), and COX-2 in adolescent patients of CFS (Brenu et al., 2014; Deumer et al., 2021). In the meantime, the miRNA expression profiles could be used to evaluate the exercise-induced changes in the expression of miRNAs involved in CFS (Cheema et al., 2020). Although the molecular mechanisms underlying the chronic fatigue syndrome have been extensively investigated, the studies of the regulatory functions of miRNAs involved in the pathological mechanism and therapeutic treatment of EIF are sparse. For example, Tung et al. have reported the inextricably linked exercise-related metabolism with miRNAs in mice with EIF generated by swimming and running (Tung et al., 2019). Furthermore, miRNAs are revealed to play critical roles in the regulation of gene expression and development of fatigue (Cui et al., 2016). To date, the molecular mechanisms underlying EIF with the involvement of the regulatory functions of miRNAs, the use of miRNAs as the potential therapeutic treatment targets of EIF, and the treatment of EIF based on various types of clinical regimes, including tea polyphenols and fruit extracts of *L. ruthenicum*, remain unclear (Yang et al., 2021). Numerous studies have revealed the significant inflammatory response to EIF, i.e., increased levels of energy substances, accelerated removal of metabolic products, and alleviated immune system disorders (Pedersen et al., 2004; Zhang et al., 2010; Smith et al., 2011). It is well known that during the oxidative stress caused by the exhaustive exercise, the levels of biochemical indices, e.g., lactate dehydrogenase (LDH) and superoxide dismutase (SOD) involved in the elimination of metabolites generated from fatigue, are significantly altered, ultimately alleviating the EIF, while the regulatory mechanisms underlying the involvement of LDH and SOD in the response to EIF are extensively explored (Armstrong et al., 1983; Radak et al., 2008; Malaguti et al., 2009; Popovic et al., 2012). Specifically, LDH metabolizes the lactic acid generated in a large amount by glycolysis during exercise to reduce the muscle soreness and to accelerate the alleviation of fatigue, while SOD is commonly considered as one of the indicators to evaluate antioxidant capacity, playing an important role in the oxidation of the body (Zhang et al., 2019; Tomazoni et al., 2019). Furthermore, after the high-intensity exercise, the serum levels of various types of cytokines, e.g., TNF-α, IL-1β, IL-2, and IL-6, are changed, revealing the oxidative stress and inflammatory response to EIF (Ostrowski et al., 1999; Starkie et al., 2000; Steinacker et al., 2004; Radak et al., 2008; Liu et al., 2017; Eaton-Fitch et al., 2021). To date, the alterations of the serum levels of these cytokines and their regulatory response to EIF are not known in mice treated with the fruit extracts of *L. ruthenicum*.

In this study, we investigated the therapeutic effects of tea polyphenols (i.e., used as the positive control) and the water extracts of *L. ruthenicum* fruits on EIF in mouse models established by swimming training method (Wang and Zhou, 2009; Xianchu et al., 2018) using the biochemical analysis and the high-throughput RNA sequencing analysis. Our goals were to validate the regulatory effects of tea polyphenols and fruit extracts of *L. ruthenicum* in the treatment of EIF in mouse models by determining the contents of a group of fatigue-related biomarkers, including LDH, SOD, TNF-α, IL-1β, IL-2, and IL-6, and identifying the fatigue-related miRNAs via high-throughput RNA sequencing technology to reveal the potential therapeutic targets for the clinical treatment of EIF. Our study was novel in that the molecular mechanisms regulating the therapeutic effects of tea polyphenols and fruit extracts of *L. ruthenicum* on the treatment of EIF in mice were explored with the involvement of miRNAs. Our study provided novel evidence to support the further agricultural development of *L. ruthenicum* and investigations and applications of both tea polyphenols and fruit extracts of *L. ruthenicum* in the treatment of EIF in humans, including the professional athletes.

## 2 Materials and methods

### 2.1 Plants materials and chemicals

Tea polyphenols (with purity of 98%) were purchased from NANJING DASF Bio-Technology Co., Ltd. (Beijing, China). Solutions of tea polyphenols fed to mice at a dose of 0.2 mg/g (based on animal body weight), 0.4 mg/g, and 0.8 mg/g, respectively, were made in a beaker with the appropriate amount of distilled water added, stirred continuously with a glass bar, and kept in a refrigerator (4°C) for use. Due to their strong antioxidant and anti-inflammatory effects as well as inhibition of chronic diseases, including EIF (Brown, 1999; Ye et al., 2009; Hadi et al., 2017; Miyata et al., 2019; Xing et al., 2019; Ohishi et al., 2021), the tea polyphenols were chosen to treat the mouse models of EIF as a type of positive control to evaluate the effects of fruit extracts of *L. ruthenicum* on the treatment of EIF. Fresh fruits of *L. ruthenicum* were purchased from Qinghai Moge Trading Co., Ltd. (Qinghai, China). Water extraction of these fruits was performed as follows: a total of 50 g fruits were added to a beaker filled with 1,000 mL distilled water and set still for 2 h, then the beaker was kept on an electric stove for extraction, stirred continuously with a glass bar. The concentration of the original liquid extraction was adjusted to 0.5 g/mL, which was then diluted to 0.05, 0.10, and 0.15 g/mL, respectively, and stored in a refrigerator (4°C) for use.

### 2.2 Experimental animals

A total of 80 specific pathogen free (SPF) Kunming mice (40 male and 40 female) with an average body weight of 20 ± 2 g were purchased from Liaoning Changsheng Agricultural Science and Technology Co., Ltd. (Shenyang, China). The selection of this type of mice was based on the previous studies on mice with EIF (Jinchun and Jie, 2011; Zhou et al., 2012; Liu et al., 2014; Yang et al., 2020). The mice were kept in cages at a room temperature of 23ºC ± 1°C with a photoperiod cycle of 12-h light (6:00 a.m. to 6:00 p.m.) and 12-h dark (6:00 p.m. to 6:00 a.m.). Food and water were available to the mice during the entire course of the experiments. The mice were treated in compliance with the principles of laboratory animal care and the guide for the care and use of laboratory animals approved by the Ethics Committee of Jilin University (approval # 2018SY0602). During the week of adaptive feeding, the mice were trained twice to accustom themselves to swimming for 5 min per time on day 2 and day 5, respectively. All mice were successful in learning to swim and were then randomly and evenly divided into 8 groups, including one model control group (MD) and seven experimental treatment groups, i.e., tea polyphenols were given at a dose of 0.2 mg/g (based on animal body weight) to the mice in the low-dose group (TPL), 0.4 mg/g in the medium-dose group (TPM), and 0.8 mg/g in the high-dose group (TPH), respectively, while the water extracts of *L. ruthenicum* fruits were given at a dose of 0.05 mg/g, 0.1 mg/g, 0.2 mg/g, and 0.5 mg/g, to mice in the low-dose group (LRL), medium-dose group (LRM), high-dose group (LRH), and super high-dose group (LRSH), respectively. The selections of these dosages were based on previous studies (Du, 2012; Yang et al., 2018) and the results of our pre-experiments. The animals were fed with either tea polyphenols or fruit extracts of *L. ruthenicum* using oral gavage needles. The same volume of distilled water was given to mice in the model control group. All animals were treated once a day with body weights recorded daily for a total of 28 days of the swimming experiments (below).

### 2.3 Swimming experiments

The swimming experiments with the mice were designed based on previous studies on the animal models of EIF (Jung et al., 2007; You et al., 2012; Chi et al., 2015), with some minor modifications. During the 28-day swimming experiments, the tea polyphenols or fruit extracts of *L. ruthenicum* were given once a day to the mice in the seven treatment groups for a total of four consecutive cycles, each cycle of 7 days, with the daily dosage for each mouse converted based on 0.1 mL/20 g. The same volume of distilled water was given to mice in the control group. Body weights of all mice were recorded daily. At the end of each cycle, the dosage was recalculated based on the body weights of mice for the next cycle. The swimming experiments were performed in a water bath with a length of 100 cm, 75 cm in width, and filled with water up to 60 cm in depth, at 32°C ± 1°C (Huang et al., 2012; Wu et al., 2013; Huang et al., 2014). The swimming experiments began 30 min after the treatments of either tea polyphenols or fruit extracts of *L. ruthenicum* every day, with the swimming time of 5, 10, 15, and 20 min in the four consecutive cycles, respectively. On the 28th day of the swimming experiments, the mice performed a single exhaustive swimming experiment with each mouse loaded with a weight (5% of its body weight) made of lead attached to the tail. The amount of time taken for each mouse to reach exhaustion was recorded. The exhaustion was defined as the evident loss of coordinated movements and failure to return to the water surface within at least 5 s (Jung et al., 2004; Shin et al., 2006; Sugino et al., 2007; Nozawa et al., 2009).

### 2.4 Biochemical analysis

One hour after the exhaustive swimming experiments, three mice were randomly chosen from each of the eight groups of mice (i.e., for a total of nine mice and 12 mice for the experimental groups treated with tea polyphenols and fruit extracts of *L. ruthenicum*, respectively) for further biochemical analyses of six fatigue-related factors, including LDH, SOD, IL-1β, IL-2, IL-6, and TNF-α, which were chosen due to their direct involvement in the pathogenesis of EIF (Liu et al., 2017; Zhang et al., 2019; Tomazoni et al., 2019). The contents of these biomarkers were determined to assess the effectiveness of tea polyphenols and fruit extracts of *L. ruthenicum* on the treatment of mice with EIF. The blood sample (∼1.5–2.0 mL) was collected from the left eyeball of each of the mice without decoagulant, cooled for about 2 h at 4°C, and then centrifugated at 1,000 × *g* and 4°C for 15 min to collect the serum in the supernatant (∼0.6–0.8 mL). The contents of a group of six fatigue-related factors, i. e., LDH, SOD, IL-1β, IL-2, IL-6, and TNF-α in serum samples, were determined by following the recommended procedures provided with the enzyme-linked immunosorbent assay (ELISA) kits (Jiangsu Mei Biao Biological Technology Co., Ltd., Jiangsu, China).

### 2.5 High-throughput RNA sequencing and MicroRNA analyses

In order to explore the molecular mechanisms regulating the therapeutic effects of tea polyphenols and fruit extracts of *L. ruthenicum* on the treatment of EIF in mice, one representative serum sample was randomly selected from each of the eight groups of mice (i.e., for a total of 3 mice and 4 mice for the experimental groups treated with tea polyphenols and fruit extracts of *L. ruthenicum*, respectively) after the 28-day swimming experiments were completed to identify the miRNAs with differential expressions in the treatment groups of mice in comparison to the model control group based on the high-throughput RNA sequencing technology (BGISEQ-500, BGI, Shenzhen, China) (Mortazavi et al., 2008; Wang et al., 2009). The raw reads of miRNAs were processed according to the procedures recommended by the manufacturer’s instructions to obtain the clean reads. The miRNAs were detected and annotated using Rfam version 12.2 (https://rfam.xfam.org/; accessed on 25 April 2019). The procedures of total RNA extraction as well as the preparations and annotations of clean tags of miRNAs were largely the same as those previously described (Li et al., 2021). Briefly, to extract the total RNA, a total of 200 μL serum sample were added with 1 mL QIAzol lysis reagent, vortexed well, and incubated at room temperature for 5 min. Then, a total of 200 μL chloroform/isoamyl alcohol (24:1; V/V) were added to the sample, vortexed vigorously for 15 s, and incubated at room temperature for 2–3 min. The samples were centrifuged at 12,000 × *g* and 4°C for 8 min to collect the supernatant (5427R, Eppendorf Co., Ltd., Beijing, China), added with absolute ethanol twice the volume of the supernatant, and the mixture was column purified and washed once with 700 μL RWT buffer and washed twice with 500 μL RPE buffer (QIAgen, Beijing, China). The samples were centrifuged at 12,000 × *g* and room temperature for 2 min with the purification column removed and replaced with a new collection tube, added with 20 μL RNA free water, and incubated at room temperature for 1 min. Finally, the mixture was centrifuged at 12,000 × *g* and room temperature for 2 min to elute RNA. The concentrations of RNAs were determined with an Agilent 2,100 Bioanalyzer (Agilent Technologies Co., Ltd., Beijing, China). The libraries for high-throughput miRNA sequencing were constructed based on the segments of 18–30 nt separated by PAGE gels (4–30 ssRNA Ladder Marker, TAKARA). The connections of both 3′ and 5′ adaptor systems and the RT-PCR analysis were performed by following the manufacturer’s procedures. The double stranded PCR products were denatured and then circularized by the splint oligo sequence to generate the single strand circle DNAs (ssCir DNAs), which were formatted as the final library. The final library was validated by the Agilent 2,100 Bioanalyzer and amplified with phi29 to generate the DNA nanoball (DNB) with more than 300 copies of one molecule. The DNBs were loaded into the patterned nanoarray to generate the single end 50 bp reads based on the combinatorial Probe-Anchor Synthesis (cPAS). The raw data were processed to generate the clean tags based on the manufacturer’s instructions. The clean tags were mapped onto the genome of *Mus musculus* with the reference version of GCF_000001635.26_GRCm38. p6 of the BioProject PRJNA20689 at the National Center for Biotechnology Information database (https://www.ncbi.nlm.nih.gov/assembly/; accessed on 21 April 2019) and further annotated based on miRbase database (http://www.mirbase.org/; accessed on 21 April 2019) and Rfam database (https://rfam.xfam.org/; accessed on 21 April 2019) with Bowtie2 (Langmead et al., 2009).

### 2.6 Identification and analysis of differentially expressed MicroRNAs

The expression levels of miRNAs were calculated by counting the absolute numbers of molecules using unique molecular identifiers (Kivioja et al., 2011), while the differential expression analysis was performed based on the PossionDis (Audic and Claverie, 1997) with the false discovery rate (FDR) ≤ 0.001 and the absolute value of |log_2_(Fold Change)| ≥ 1 as the default threshold to determine the significant difference of expression. The target genes of differentially expressed miRNAs were identified by both MiRanda (John et al., 2004) and TargetScan (Agarwal et al., 2015). The functions of target genes were further annotated based on the Gene Ontology (GO; http://www.geneontology.org/; accessed on 25 April 2019) and the Kyoto Encyclopedia of Genes and Genomes (KEGG; http://www.genome.jp/kegg/; accessed on 25 April 2019) databases with the *p*-value ≤0.05 as a threshold corrected based on the Bonferroni method (Abdi, 2007).

### 2.7 Statistical analyses

All statistical analyses were performed using Graphpad Prism 8 statistical software with data presented as mean ± standard deviation (SD). One-way ANOVA was performed to determine the significant difference among groups with the *p*-value ≤0.05 considered as statistically significant.

## 3 Results

### 3.1 Effects of tea polyphenols and fruit extracts of *Lycium ruthenicum* on body weight and exhaustive swimming of mice with exercise-induced fatigue

**FIGURE 1:**
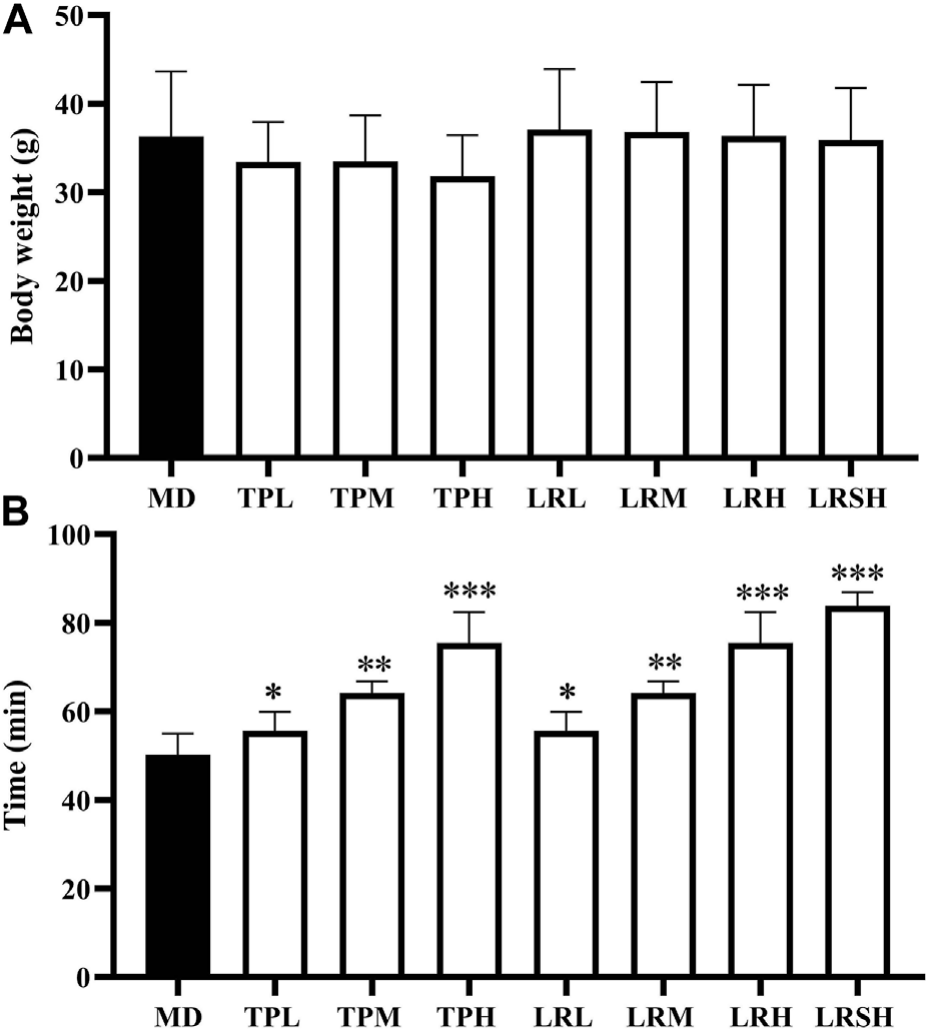
Effect of tea polyphenol and fruit extracts of *Lycium ruthenicum* on body weight change **(A)** and exhaustive swimming duration **(B)** in eight groups of mice with exercise-induced fatigue (EIF). The mice in the model control groups (MD) are given distilled water, while the experimental groups are treated with either tea polyphenols of low-dose (TPL), medium-dose (TPM), and high-dose (TPH) or fruit extracts of *L. ruthenicim* of low-dose (LRL), medium-dose (LRM), high-dose (LRH), and super high-dose (LRSH), respectively. Significant difference is determined by the paired Student’s *t*-test with *p* < 0.05 (*), *p* < 0.01 (**), and *p* < 0.001 (***), respectively.

Compared with the model control group, the body weights of mice in the seven experimental groups fed with either tea polyphenols or fruit extracts of *L. ruthenicum* showed no significant difference in 28 days (Figure 1A), whereas the significant increases were observed in the exhaustive swimming duration in all experimental groups of mice (Figure 1B). Both treatments of tea polyphenols and fruit extracts of *L. ruthenicum* showed comparable effects on the exhaustive swimming duration of mice with EIF. These results strongly suggested that the establishment of the mouse models of EIF in our study was successful, while both tea polyphenols and fruit extracts of *L. ruthenicum* could significantly alleviate the EIF in mice.

### 3.2 Effects of tea polyphenols and fruit extracts of *Lycium ruthenicum* on the oxidative stress and anti-inflammatory response in mice with exercise-induced fatigue

**FIGURE 2:**
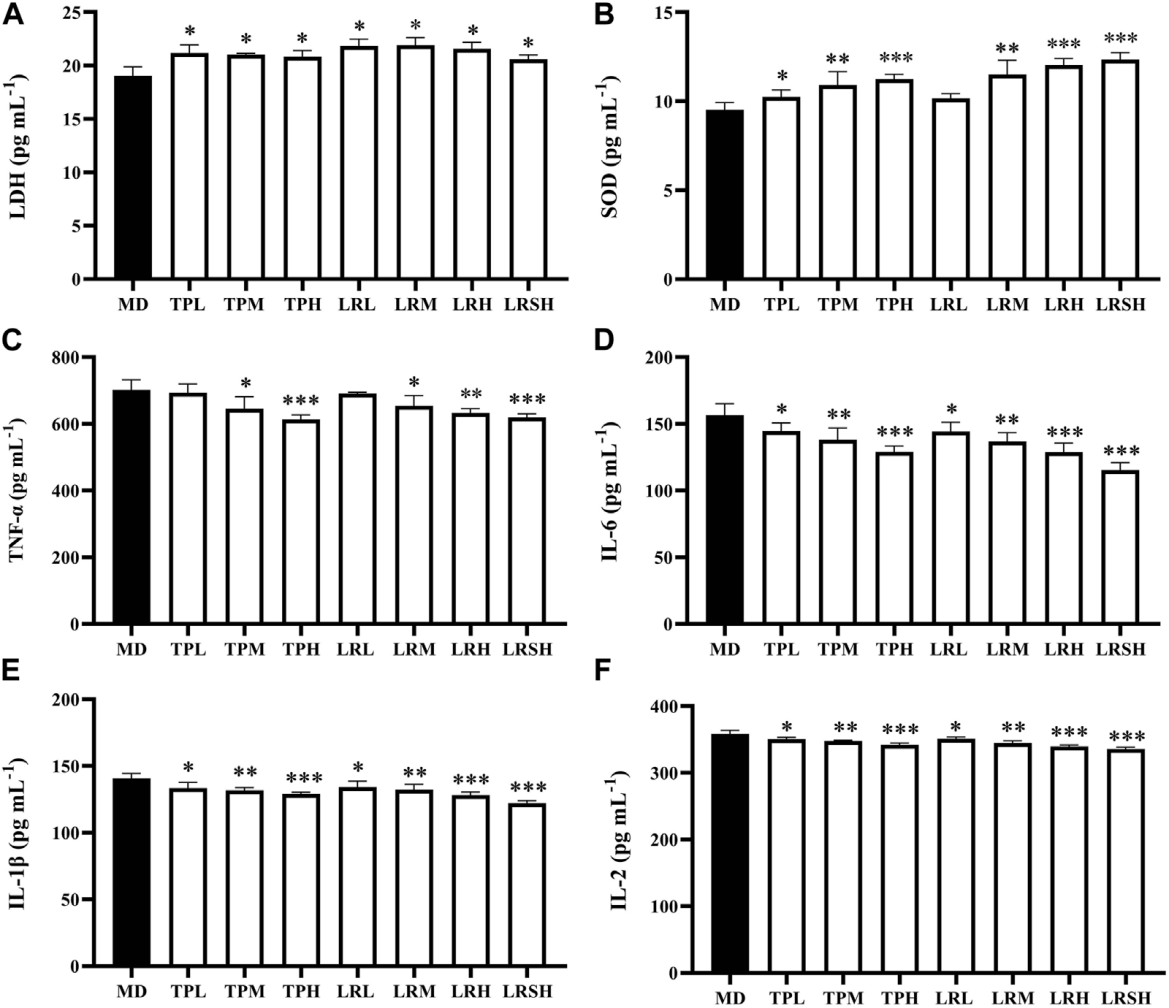
Effects of tea polyphenols and fruit extracts of *Lycium ruthenicum* on the contents of two oxidative stress related biomarkers, i.e., **(A)** lactate dehydrogenase (LDH) and **(B)** superoxide dismutase (SOD), and four types of inflammatory cytokines, i.e., **(C)** tumor necrosis factor-α (TNF-α), **(D)** interleukin-6 (IL-6), **(E)** interleukin-1β (IL-1β), and **(F)** interleukin-2 (IL-2), in serum of eight groups of mice with exercise-induced fatigue (EIF). The model control groups (MD) are treated with distilled water, while the experimental groups are treated with either tea polyphenols of low-dose (TPL), medium-dose (TPM), and high-dose (TPH) or fruit extracts of *L. ruthenicum* of low-dose (LRL), medium-dose (LRM), high-dose (LRH), and super high-dose (LRSH), respectively. Significant difference is determined by the paired Student’s *t*-test with *p* < 0.05 (*), *p* < 0.01 (**), and *p* < 0.001 (***), respectively.

To explore the preventive effects of tea polyphenols and fruit extracts of *L. ruthenicum* on the oxidative stress in mice with EIF, the contents of two types of oxidative stress related antioxidant enzymes (i.e., LDH and SOD) were determined in serum of eight groups of mice after they performed the exhaustive swimming experiments. The results showed that after 28 days of treatment, the contents of LDH in both TPM and TPH groups as well as the LRM, LRH, and LRSH groups were significantly decreased compared with the model control group (Figure 2A), while the contents of SOD were significantly increased in most of the treatment groups of both tea polyphenols and fruit extracts of *L. ruthenicum* except for the LRL group (Figure 2B).

To explore the effects of tea polyphenols and fruit extracts of *L. ruthenicum* on the anti-inflammatory response in mice with EIF, the contents of four types of inflammatory cytokines (i.e., TNF-α, IL-6, IL-1β, and IL-2) were determined in serum of mice after the exhaustive swimming experiments (Figures 2C–F). The results showed that in 28 days after the treatments of either tea polyphenols of fruit extracts of *L. ruthenicum* at varied doses in mice with EIF, the contents of all four types of inflammatory cytokines were significantly decreased in most of the treatment groups of mice except for the contents of TNF-α in both TPL and LRL groups in comparison to the model control group of mice. Both treatments of tea polyphenols and fruit extracts of *L. ruthenicum* showed comparable effects on the contents of these cytokines in mice with EIF. These results evidently revealed the anti-inflammatory responses and reduction of oxidative stress in mice with EIF treated with either tea polyphenols or fruit extracts of *L. ruthenicum*, suggesting that the treatments of both tea polyphenols and fruit extracts of *L. ruthenicum* alleviated the fatigue by regulating the inflammatory responses in mice.

### 3.3 Characteristics of microRNAs and differentially expressed microRNAs in mice with exercise-induced fatigue

To explore the regulatory mechanisms underlying the therapeutic effects of tea polyphenols and fruit extracts of *L. ruthenicum* on EIF, the high-throughput RNA sequencing was performed to identify the miRNAs involved in the molecular response to the EIF in mice treated with either tea polyphenols or fruit extracts of *L. ruthenicum*. The clean tags of small RNAs sequenced based on BGISEQ-500 technology were generated for further analyses (Supplementary Table S1). These results revealed that the lengths of a total of 1,976 miRNAs ranged from 15 to 27 bases with the most abundant miRNAs of 21, 22, and 23 bases with a total of 436, 881, and 323 miRNAs, respectively.

**FIGURE 3:**
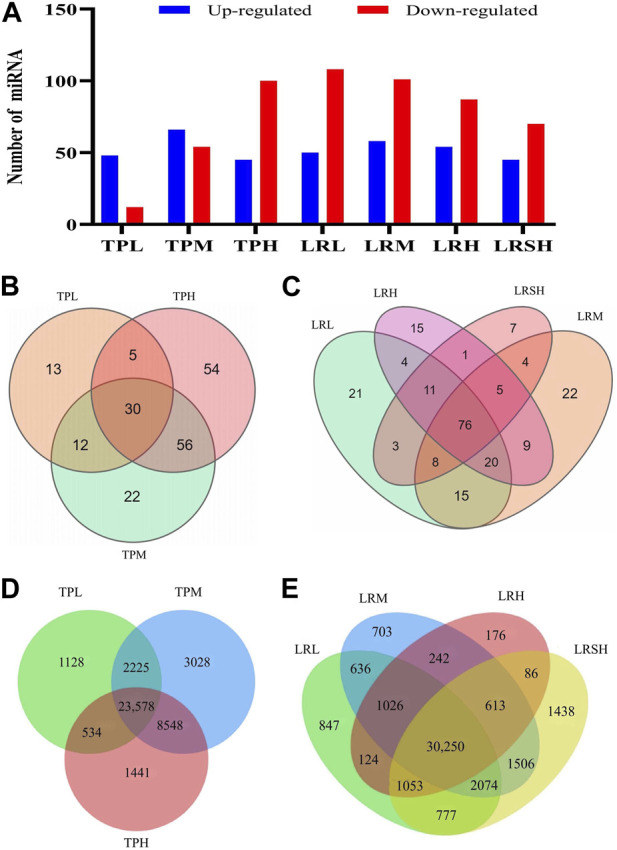
Analyses of differentially expressed microRNAs in the seven experimental groups of mice treated with either tea polyphenols with low-dose (TPL), medium-dose (TPM), and high-dose (TPH) or fruit extracts of *Lycium ruthenicum* with low-dose (LRL), medium-dose (LRM), high-dose (LRH), and super high-dose (LRSH), in comparison to the model control group of mice with exercise-induced fatigue (EIF). **(A)** Differentially expressed microRNAs (upregulated and downregulated) in the seven experimental groups of mice. **(B)** Differentially expressed microRNAs in the three experimental groups of mice treated with tea polyphenols. **(C)** Differentially expressed microRNAs in the four experimental groups of mice treated with fruit extracts of *L. ruthenicum*. **(D)** Target genes predicted by both TargetScan and miRanda of the differentially expressed microRNAs identified in three groups of mice with EIF treated with tea polyphenols. **(E)** Target genes predicted by both TargetScan and miRanda of the differentially expressed microRNAs identified in four groups of mice with EIF treated with fruit extracts of *L. ruthenicum*.

Differentially expressed miRNAs were identified based on FDR ≤0.001 and |log_2_(Fold Change)| ≥ 1 in the seven experimental groups of mice with EIF in comparison to the model control group to evaluate the variation in the expression levels of miRNAs in response to the treatments of tea polyphenols and fruit extracts of *L. ruthenicum* (Figure 3A). The heatmaps of miRNAs in eight groups of mice and the differentially expressed miRNAs in the seven experimental groups of mice in comparison to the model control group of mice were presented in the hierarchical clusters (Supplementary Figures S1, S2). These results showed that the highest number of a total of 159 differentially expressed miRNAs were revealed in the LRM group, while the TPL, LRSH, and TPH groups showed relatively smaller numbers of differentially expressed miRNAs of 60, 115, and 120, respectively (Figure 3A). The highest numbers of upregulated miRNAs of 66 and 58 were obtained in the TPM and LRM groups, respectively, while the lowest number of upregulated miRNAs of 45 was identified in both the TPH and LRSH groups. The highest number of downregulated miRNAs of 108 was revealed in the LRL group, while the lowest number of downregulated miRNAs of 12 and 54 were revealed in TPL and TPM groups, respectively. These results clearly indicated that the miRNAs were involved in the molecular response to the treatment of tea polyphenols and fruit extracts of *L. ruthenicum* in mice with EIF.

In comparison to the model control group, a total of 30, 90, and 115 differentially expressed miRNAs were identified in the TPL, TPM, and TPH groups, respectively, while a total of 13, 22, and 54 miRNAs were uniquely identified in the TPL, TPM, and TPH groups, respectively (Figure 3B). A total of 30 miRNAs were commonly identified in the three groups of mice treated with tea polyphenols. In comparison to the control group, a total of 82, 83, 65, and 39 differentially expressed miRNAs were identified in the LRL, LRM, LRH, and LRSH groups, respectively, while a total of 21, 22, 15, and 7 miRNAs were uniquely identified in the LRL, LRM, LRH, and LRSH groups, respectively (Figure 3C). A total of 76 miRNAs were commonly identified in the four groups of mice treated with fruit extracts of *L. ruthenicum*. A total of 23 miRNAs (21 upregulated and 2 downregulated) were commonly identified between the 30 miRNAs identified as the common set of miRNAs in the three groups of mice treated with tea polyphenols and the 76 miRNAs identified as the common set of miRNAs in the four groups mice treated with fruit extracts of *L. ruthenicum* (Table 1).

**TABLE 1:** A total of 23 differentially expressed microRNAs (21 upregulated and 2 downregulated) commonly identified between the three experimental groups of mice treated with tea polyphenols and four experimental groups of mice treated with fruit extracts of *Lycium ruthenicum*.

Expression pattern	Differentially expressed miRNA
Upregulation	let-7c-2-3p, miR-106b-5p, miR-130a-3p, miR-141-3p, miR-142a-3p, miR-142a-5p, miR-144-3p, miR-144-5p, miR-152-3p, miR-15b-3p, miR-19b-3p, miR-194-5p, miR-1a-1-5p, miR-20a-5p, miR-24-2-5p, miR-29a-3p, miR-29b-3p, miR-30a-5p, miR-30e-5p, miR-499-5p, miR-500-3p
Downregulation	let-7c-5p, miR-486a-3p

### 3.4 Target genes of differentially expressed microRNAs in mice with exercise-induced fatigue

To further investigate the potential biological functions of differentially expressed miRNAs, the target genes of these miRNAs in the three groups of mice treated with tea polyphenols and four groups of mice treated with fruit extracts of *L. ruthenicum* were predicted by TargetScan and miRanda. A total of 40,482 target genes of the differentially expressed miRNAs in the three groups of mice treated with tea polyphenols were identified with a total of 23,578 genes commonly detected in these three groups of mice (Figure 3D), while a total of 41,551 target genes of the differentially expressed miRNAs in the four groups of mice treated with fruit extracts of *L. ruthenicum* were predicted with a total of 30,250 genes commonly identified in these four groups of mice (Figure 3E). These variations in the numbers of target genes suggested that the therapeutic effects of tea polyphenols and fruit extracts of *L. ruthenicum* on EIF in mice were probably regulated by varied molecular mechanisms. Future studies are necessary to investigate the explicit functions of these target genes in the pathology and pathogenesis of EIF and the pharmacological mechanisms of tea polyphenols and fruit extracts of *L. ruthenicum* on alleviating EIF in mice.

### 3.5 Enrichment analyses of target genes of differentially expressed microRNAs

Both GO annotation and KEGG enrichment analyses were performed to investigate the functions of the target genes of the differentially expressed miRNAs identified in the seven experimental groups of mice treated with either tea polyphenols or fruit extracts of *L. ruthenicum*.

**FIGURE 4:**
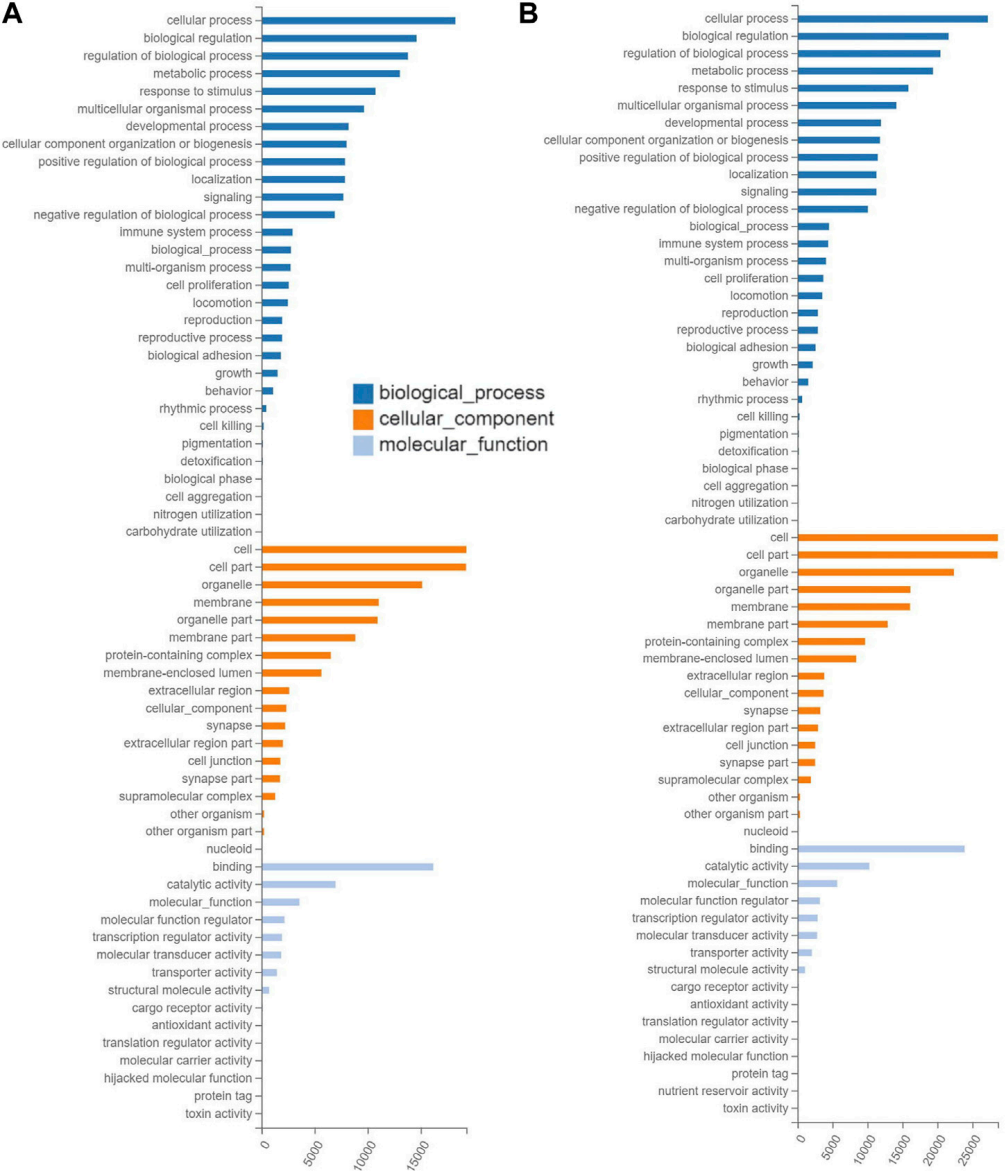
Functional annotations based on the Gene Ontology (GO) database of the target gene of differentially expressed microRNAs identified in the experimental groups of mice treated with tea polyphenols of low-dose **(A)** and with fruit extracts of *Lycium ruthenicum* of low-dose **(B)** in comparison with the model control group fed with distilled water, respectively, showing the number of differentially expressed microRNAs (*X*-axis) annotated in the three groups of GO terms (i.e., biological process, cellular component, and molecular function).

The results of GO annotations showed that the similar numbers of target genes in the ranges of 22,089–29,448, 22,163–29,502, and 21,846–29,093 were annotated in the three categories of GO terms, i.e., biological process, cellular component, and molecular function, respectively, in the three groups of mice treated with tea polyphenols (Figure 4A; Supplementary Figure S3), while in the four groups of mice treated with fruit extracts of *L. ruthenicum*, a total of 29,727–33,098, 29,771–33,147, and 29,354–32,677 target genes were annotated in three categories of GO database, respectively (Figure 4B; Supplementary Figure S4). These results revealed similar patterns of gene annotations in the seven experimental groups of mice, with the greatest number of genes annotated to the category of biological process of GO terms, while the category of molecular function contained the least number of genes annotated. In the category of biological process of GO database, the top five GO terms with the highest numbers of functional genes annotated (14,148–27,205) included cellular process, biological process, regulation of biological process, metabolic process, and response to stimulus, while the other 25 GO terms were annotated with genes ranging from 3 to 14,135. In the category of cellular component in GO database, the top three GO terms (i.e., cell, cell part, and organelle) were annotated with the highest numbers of genes ranging from 15,131 to 28,648, while the other 15 GO terms were annotated with genes ranging from 51 to 16,140. In the category of molecular function, two GO terms (i.e., binding and catalytic activity) were revealed to contain the largest number of genes annotated (6,980–23,893), while the other 11 GO terms were annotated with genes ranging from 1 to 5,684.

The results of GO annotations of target genes of differentially expressed miRNAs revealed that these miRNAs were significantly responsive to EIF in mice treated with tea polyphenlos and fruit extracts of *L. ruthenicum* and were involved in many molecular processes (Supplementary Table S2), mainly including skeletal muscle regeneration, tissue development, and system development (GO:0014905, GO:0007519, and GO:0001501), myoblast fusion (GO:0007520), cell proliferation and differentiation (GO:0008284 and GO:0045596), apoptotic process (GO:0043066), motor activity (GO:0003774), and T cell differentiation and proliferation (GO:0045580 and GO:0042130).

**FIGURE 5:**
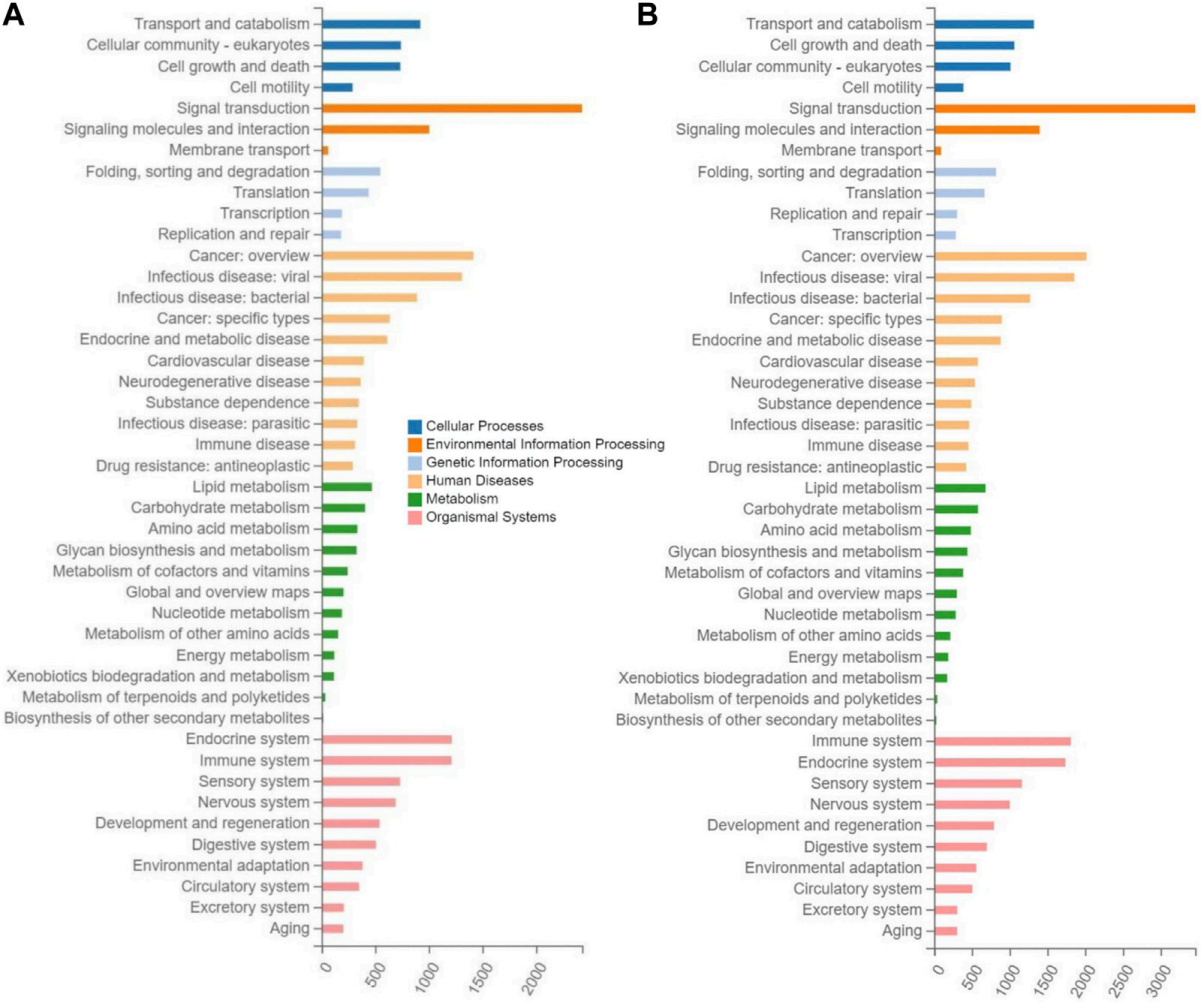
Metabolic pathway enrichment analysis based on the Kyoto Encyclopedia of Genes and Genomes (KEGG) database of the target genes of the differentially expressed microRNAs identified in the experimental groups of mice treated with tea polyphenols of low-dose **(A)** and with fruit extracts of *Lycium ruthenicum* of low-dose **(B)**, in comparison with the model control group fed with distilled water, respectively, showing the number of differentially expressed microRNAs (*X*-axis) enriched in the six categories of metabolic pathways (i.e., cellular process, environment information processing, genetic information processing, human diseases, metabolism, and organismal systems) in the KEGG database.

The enrichment analyses of the target genes of differentially expressed miRNAs were performed based on the KEGG database to reveal the metabolic pathways involved in the molecular mechanisms underlying the therapeutic effects of tea polyphenols and fruit extracts of *L. ruthenicum* on mice with EIF. The results revealed that similar patterns of metabolic pathways were observed among the seven experimental groups of mice with EIF, showing that a total of 44 KEGG pathways were enriched in a total of six categories of KEGG database, including human diseases, organismal systems, environmental information processing, cellular process, metabolism, and genetic information processing (Figure 5; Supplementary Figures S5, S6). The highest numbers of target genes (ranging from 3,862 to 5,717) were enriched in the category of organismal systems, followed by the categories of human diseases (with annotated genes ranging from 3,421 to 4,946) and environment information processing (with annotated genes ranging from 3,006 to 4,295), while the lowest numbers of target genes (ranging from 1,307 to 1,996) were observed in the category of genetic information processing, and the categories of metabolism and cellular process were annotated with genes ranging from 1,908 to 2,799 and from 2,154 to 3,043, respectively.

**FIGURE 6:**
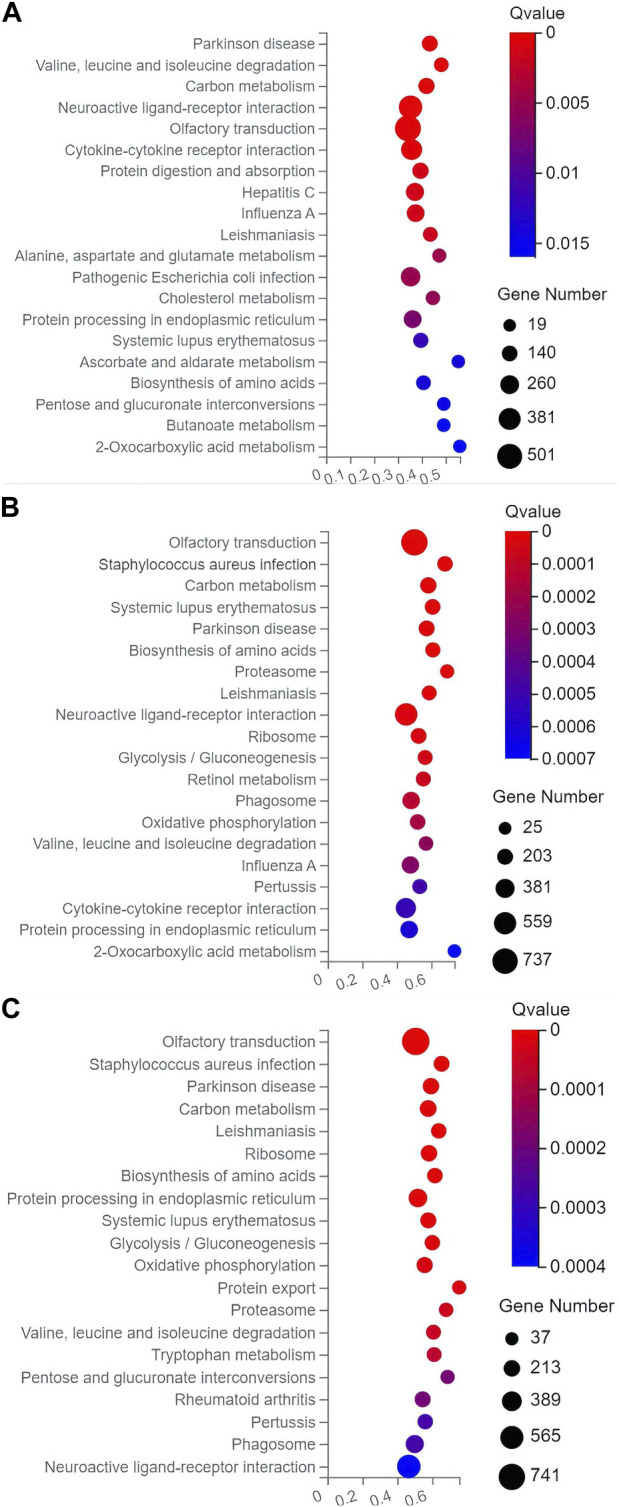
Scatter plots of the top 20 categories of enriched metabolic pathways based on the Kyoto Encyclopedia of Genes and Genomes (KEGG) database of the target genes of the differentially expresses microRNAs identified in the three groups of mice treated with tea polyphenols of low-dose **(A)**, medium-dose **(B)**, and high-dose **(C)** in comparison with the model control group, respectively, showing the number of genes annotated in each metabolic pathway. The Rich Ratio (*X*-axis) is the ratio of the number of target genes of the differentially expressed microRNAs annotated in each pathway to the total number of genes annotated in the pathway. The greater value of Rich Ratio (*X*-axis) indicates the greater degree of enrichment. The Q-values are corrected *p*-value ranging from 0 to 1 with the lower Q-value representing the greater level of enrichment.

**FIGURE 7:**
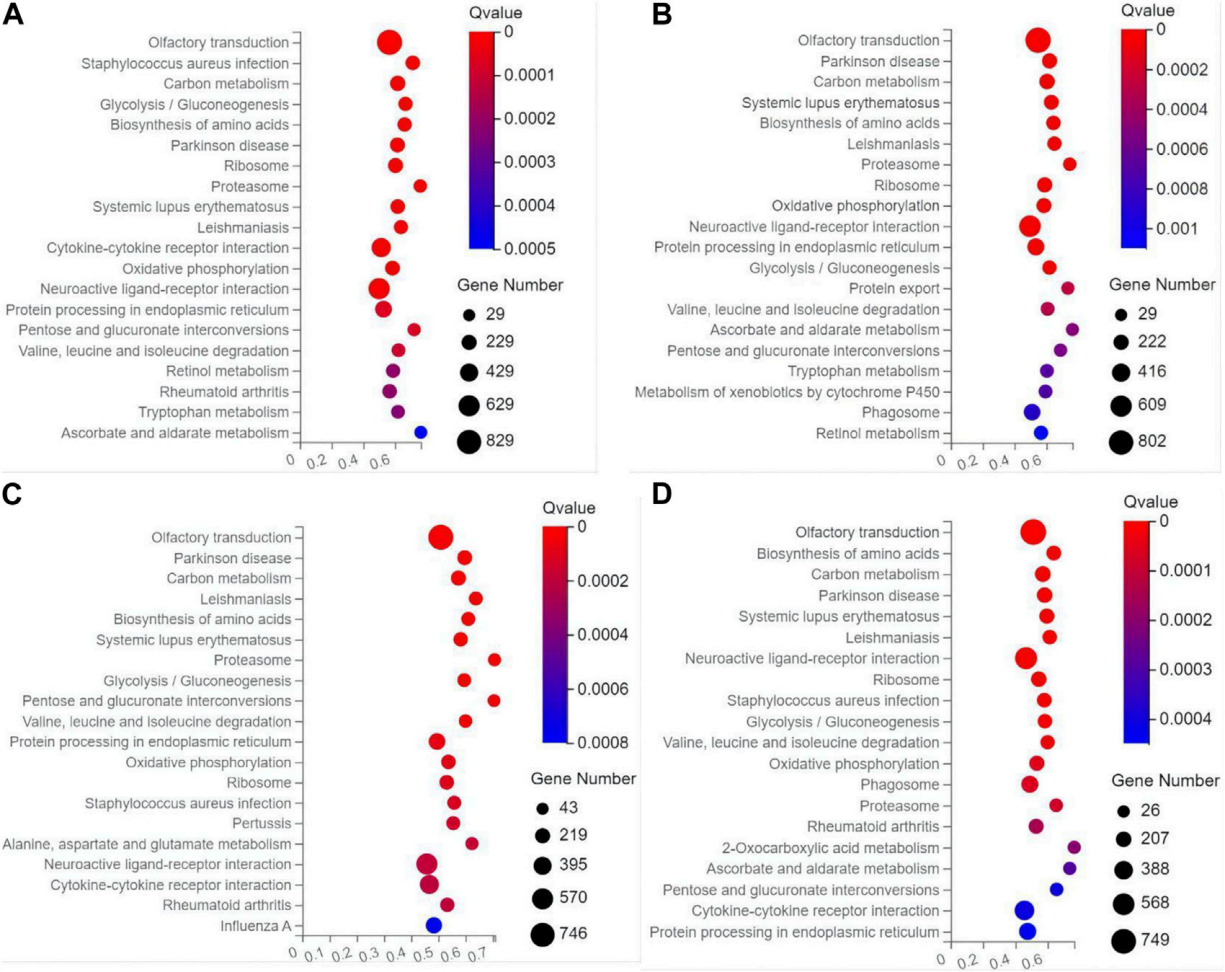
Scatter plots of the top 20 categories of enriched metabolic pathways based on the Kyoto Encyclopedia of Genes and Genomes (KEGG) database of the target genes of the differentially expresses microRNAs identified in the four groups of mice treated with fruit extracts of *L. ruthenicum* of low-dose **(A)**, medium-dose **(B)**, high-dose **(C)**, and super high-dose **(D)** in comparison with the model control group, respectively, showing the number of genes annotated in each metabolic pathway. The Rich Ratio (*X*-axis) is the ratio of the number of target genes of the differentially expressed microRNAs annotated in each pathway to the total number of genes annotated in the pathway. The greater value of Rich Ratio (*X*-axis) indicates the greater degree of enrichment. The Q-values are corrected *p*-value ranging from 0 to 1 with the lower Q-value representing the greater level of enrichment.

In the top 20 metabolic pathways enriched in the KEGG database, the pathway of olfactory transduction was enriched with a total of ∼500–800 functional genes in the seven experimental groups of mice, followed by the pathway of neuroactive ligand-receptor interaction annotated with a total of ∼300–600 genes and the pathway of cytokine-cytokine receptor interaction enriched with ∼400–500 genes (Figures 6, 7). It was noted that the cytokine-cytokine receptor interaction was not one of the top 20 pathways enriched in the TPH and LRM groups of mice. Further studies are needed to explore the functions of these genes and pathways, in particular, the genes annotated in the pathways of olfactory transduction and neuroactive ligand-receptor interaction, in the molecular responses to EIF in mice treated with tea polyphenols and fruit extracts of *L. ruthenicum*.

## 4 Discussion

### 4.1 Effects of tea polyphenols and fruit extracts of *Lycium ruthenicum* on the oxidative stress in mice with exercise-induced fatigue

Our results clearly demonstrated that both the tea polyphenols and fruit extracts of *L. ruthenicum* showed evident antioxidant effects on EIF in mice. Further studies are needed to explicitly determine the dosage of either tea polyphenols or fruit extracts of *L. ruthenicum* in their applications in the treatment of EIF. The strenuous exercises generally cause the accumulation of a large amount of lactic acid, ultimately generating the fatigue in the body*.* LDH is a key glycolytic enzyme in the anaerobic metabolism of sugar to catalyze the conversion between pyruvate and lactic acid. Therefore, the content of LDH could be served as an important index to measure the levels of anaerobic metabolism in the body, in particular, reflecting the metabolism rate of the clearance of lactic acid in muscles, i.e., the enhanced activities of LDH accelerate the metabolism of excessive lactic acid in muscle, ultimately delaying the occurrence or accelerating the elimination of fatigue. Our results showed that both tea polyphenols and fruit extracts of *L. ruthenicum* of varied doses significantly decreased the levels of LDH in mice with EIF, suggesting that both tea polyphenols and fruit extracts of *L. ruthenicum* effectively increased the metabolism of lactic acid products, reducing the accumulation of lactic acid metabolites and the concentration of lactic acid in blood, eventually accelerating the alleviation of fatigue. These results were consistent with those reported previously. For example, the LDH levels were elevated in the rat and mouse models of EIF treated with different doses of tea polyphenols (Du, 2012; Zhang et al., 2013), while the serum levels of LDH were significantly increased in mice by the treatment of fruit polyphenols extracted from *Lonicera caerulea* with different doses in 6 weeks (Liu et al., 2022).

Both the anti-fatigue and anti-oxidation effects are generally closely related in their functional metabolic pathways. The excessive oxygen free radicals generated during the prolonged and high-intensity exercises could cause oxidative damage of tissues or organs, ultimately causing fatigue in muscles. As an important antioxidant enzyme, SOD is commonly used as a biomarker for the evaluation of antioxidant capacity and oxidative damage due to its capability of scavenging oxygen free radicals in the body with the level of SOD indirectly reflecting the levels of tissue damage. Our results showed that in comparison to the model control group, the contents of SOD in serum of mice treated with either tea polyphenols or fruit extracts of *L. ruthenicum* at varied doses were significantly increased. These results were consistent with those previously reported. For example, the concentrations of SOD in serum of rats treated with varied doses of tea polyphenols for 30 days were significantly increased after the exhaustion of the treadmill experiments (Du, 2012). Furthermore, the polysaccharides extracted from the fruits of *L. ruthenicum* were revealed to change the contents of both LDH and SOD in mice with EIF, suggesting the strong anti-fatigue effects of fruit extracts of *L. ruthenicum* (Ni et al., 2013). Similarly, other medicinal plants have also been used to treat the fatigue, showing the consistent results with our findings of the content changes in both LDH and SOD caused by the treatment of both tea polyphenols and fruit extracts of *L. ruthenicum*. For example, studies have shown that root extracts of *Aralia continentalis* significantly decreased the contents of LDH and increased the contents of SOD in rats with EIF to achieve the prevention of oxidative stress (Yang D. K. et al., 2020). Furthermore, the seed extracts of *Nigella sativa* were used to significantly alter the contents of both LDH and SOD in rats with EIF, showing the anti-fatigue effects on EIF (Rahman et al., 2017). These results suggested that the treatments of both tea polyphenols and fruit extracts of *L. ruthenicum* delayed or prevented the occurrence of fatigue by eliminating the oxygen free radicals produced during the strenuous exercise, decreasing the body damage by increasing the SOD activity in serum of the body, and ultimately protecting the body from oxidative stress.

### 4.2 Effects of tea polyphenols and fruit extracts of *Lycium ruthenicum* on the anti-inflammatory response in mice with exercise-induced fatigue

Studies have shown that the acute high-intensity exercises could alter the balance of cytokines throughout the body, while many of these cytokines are cellular signals associated with oxidative stress and inflammatory responses (Liu et al., 2017). In particular, fatigue generates a series of inflammatory responses in the body, changing the levels of IL-1β, IL-2, IL-6, and TNF-α, which are the key pro-inflammatory cytokines involved in the initiation of the anti-inflammatory response to injury. For example, secreted by mononuclear macrophages, TNF-α has shown strong pro-inflammatory effects (Balzano et al., 2020), i.e., inducing the chemotaxis of monocytes and polygamous cells to infiltrate the inflammatory area, activating the vascular endothelial cells, and degranulating the neutrophils to release oxygen free radicals, lipid metabolites, lysosomal enzymes, and other mediators (Hong et al., 2021). Furthermore, the activities of IL-6 include the recruitment of inflammatory cells, inhibition of inflammatory cells, and inhibition of regulatory T cell differentiation (Tanaka et al., 2014; Libby, 2017), while the IL-1β cooperates with IL-2 to enhance the activities of natural killer cells and promote the secretion of inflammatory mediators by various inflammatory cells (Abbas et al., 2018; Das et al., 2021). Our results showed that the contents of IL-1β, IL-2, IL-6, and TNF-α were significantly decreased in serum of mice treated with either tea polyphenols or fruit extracts of *L. ruthenicum*. These results suggested that tea polyphenols and fruit extracts of *L. ruthenicum* promoted the ability of liver cells to take amino acids, simultaneously increasing the activity of natural killing cells by both IL-1β and IL-2 to provide inflammatory mediators for the body during strenuous exercise and to accelerate the elimination of fatigue. Similarly, studies have revealed the protective effects of tea polyphenols on acute EIF and inflammatory injury in rats, showing the significantly decreased levels of LDH, IL-1β, and IL-6 in serum (Liu et al., 2017). Furthermore, studies have shown that the levels of TNF-α, IL-1β, and IL-6 were significantly decreased in mice fed with high-fat diet and treated with anthocyanins of *L. ruthenicum*, suggesting the regulatory effects of the fruit extracts of *L. ruthenicum* on the inflammation in mice (Tian et al., 2021). Moreover, the levels of both serum IL-6 and TNF-α were significantly decreased by the treatment of fruit extracts of *L. ruthenicum* in rats trained with aerobic exercise, showing the regulatory effects of fruit extracts of *L. ruthenicum* on the myocardial lipid metabolism in rats with high-fat diet (Cui et al., 2020). These data evidently indicated that both tea polyphenols and fruit extracts of *L. ruthenicum* were revealed to show protective and anti-inflammatory effects on mice with EIF, confirming the therapeutic effects of tea polyphenols and fruit extracts of *L. ruthenicum* on EIF in mice. Therefore, it is extremely important to further explore the molecular mechanisms regulating the therapeutic effects of tea polyphenols and fruit extracts of *L. ruthenicum* on the clinical treatment of EIF, which provides a potentially novel and prospective medical remediation in various types of sports to alleviate or ultimately prevent the EIF in professional athletes (Bowtell and Kelly, 2019).

### 4.3 Differentially expressed microRNAs in mice with exercise-induced fatigue

To date, the molecular mechanisms regulating the therapeutic effects of tea polyphenols and fruit extracts of *L. ruthenicum* on the treatment of EIF with the involvement of miRNAs remain unclear. Our results showed for the first time that a total of 23 differentially expressed miRNAs were involved in the molecular response to the EIF in mice treated with either tea polyphenols or fruit extracts of *L. ruthenicum*, suggesting the participation of these miRNAs in the pathogenesis of EIF in mice. Previous studies showed that three out of these miRNAs, i.e., miR-29a-3p, miR-30a-5p, and miR-30e-5p, were involved in various types of medical disorders related to EIF. For example, studies have shown that the expression of miR-29a-3p among a group of nine miRNAs involved in pain and fatigue was significantly decreased in patients with fibromyalgia (Bjersing et al., 2013), while the enhanced expression of miR-29 improved the cardiac functions in rats with aerobic exercise training (Soci et al., 2011). Furthermore, studies showed that miR-29a-3p was involved in the metabolic pathways leading to myalgic encephalomyelitis/chronic fatigue syndrome (Nepotchatykh et al., 2020). These results were consistent with the findings revealed in our study, showing the significant upregulation of miR-29a-3p in response to treatment of either tea polyphenols or fruit extracts of *L. ruthenicum* in mice of EIF. Moreover, our results showed that the miR-30 family members were involved in the molecular response to the treatment of mice with EIF by either tea polyphenols or fruit extracts of *L. ruthenicum*. These results were consistent with those previously reported. For example, among a group of five miRNAs, miR-30e-5p with significant regulations was identified as a potential biomarker in regulating myasthenia gravis (i.e., clinically characterized by fatigue) or muscle homeostasis (Sabre et al., 2020), while the expression of miR-30a-5p was upregulated as an acute effect of resistance training (Vogel et al., 2019). These results were consistent with the findings revealed in our study, showing the significant up-regulations of both miR-30a-5p and miR-30e-5p in response to the treatment of either tea polyphenols or fruit extracts of *L. ruthenicum* in mice with EIF. These results suggested that the therapeutic effects of tea polyphenols on EIF in mice were probably regulated by different molecular mechanisms from those of the fruit extracts of *L. ruthenicum*. Further studies are necessary to investigate the explicit functions of these miRNAs in the pathology and pathogenesis of EIF and the pharmacological mechanisms underlying the therapeutic effects of tea polyphenols and fruit extracts of *L. ruthenicum* on the treatment of EIF in mice.

### 4.4 Annotation and enrichment analyses of target genes of differentially expressed microRNAs

Our results of GO annotation based on the target genes of differentially expressed miRNAs in mice of EIF treated with tea polyphenols and fruit extracts of *L. ruthenicum* were consistent with those reported previously, showing that the miRNA expression was involved in the functional regulation of immune cells and the occurrence of chronic fatigue syndrome (Brenu et al., 2012; Petty et al., 2016). In particular, miR-106 was involved in the expression of immune cells and inflammatory response under the condition of chronic fatigue. Furthermore, it was reported recently that the fruit extracts of polyphenols in *L. caerulea* alleviated the exercise fatigue in mice by reducing skeletal muscle cell apoptosis and increasing cell proliferation (Liu et al., 2022). These results suggested that the differentially expressed miRNAs revealed in our study were involved in many immune responses to alleviate the EIF in mice. These results were consistent with the findings revealed by the pathogenetic investigations in our study and with those previously reported (Gandevia, 2001; Cowling et al., 2008; Westerblad et al., 2010; Bossola et al., 2016; Taylor et al., 2016; Heaton et al., 2017; Burnley and Jones, 2018). Further studies are necessary to investigate the detailed regulatory functions of these miRNAs and their target genes in the occurrence of EIF and the pharmacological mechanisms of tea polyphenols and fruit extracts of *L. ruthenicum* in the alleviation of EIF in mice. Moreover, studies showed that a group of miRNAs were involved in the skeletal system development pathway (Westerblad et al., 2010), consistent with the findings revealed in our study, to significantly improve the skeletal muscle atrophy and function as well as the anti-fatigue ability in mice (Shin et al., 2020). In particular, our results showed that a group of 4 miRNAs (i.e., miR-486a-3p, miR500-3p, miR-24-2-5p, and miR-194-5p) were involved in the regulation of skeletal system development. Further studies are needed to verify the regulatory functions of these miRNAs in skeletal system development. Our results identified that both miR-130a-3p and let-7f-2-3p were involved in the glycogen and glucose metabolic pathways, which were commonly considered as the metabolic pathways involved in fatigue. Additionally, studies showed that reactive oxygen species (ROS) metabolism played an important role in exercise fatigue (Powers et al., 2020), while the fatigue and change in oxidative metabolism level were derived from the change of miRNA expression (Dickey et al., 2021). Furthermore, studies showed that the ROS production was reduced by the fruit polyphenols of *L. caerulea* via the PKC-NOX2/Nox4 pathway in mice with EIF (Liu et al., 2022). In our study, three miRNAs (i.e., miR-500-3p, miR-142a-5p, and miR-24-2-5p) were involved in the ROS metabolic pathway. These results suggested that the tea polyphenols and fruit extracts of *L. ruthenicum* alleviated the EIF by decreasing the oxidative stress in mice, further suggesting that the fruit extracts of *L. caerulea* and *L. ruthenicum* regulated the ROS pathway with varied molecular mechanisms. Further studies are needed to explicitly verify the molecular functions of these miRNAs in response to EIF in mice.

The results of the KEGG enrichment analysis revealed that many metabolic pathways were enriched by the target genes of differentially expressed miRNAs in mice of EIF treated with tea polyphenols and fruit extracts of *L. ruthenicum*, evidently suggesting that these differentially expressed miRNAs and their target genes were involved in these metabolic pathways in response to EIF in mice. These results were consistent with the results revealed by the pathogenetic investigations in our study and with those previously reported. For example, as one of the significantly expressed miRNAs, miR-30e-5p was involved in the prevention of tumor growth and angiogenesis in gallbladder cancer via the PI3K/Akt pathway (Jin et al., 2018), while the inhibition of miR-30a-5p significantly promoted proliferation, migration, invasion, epithelial-interstitial transformation, and apoptosis of NON-small cell lung cancer cells, and enhanced the PI3K/Akt expression (Wang et al., 2020; Li et al., 2022), suggesting that both miR-30a-5p and miR-30e-5p played important roles in the treatment of mice with EIF using tea polyphenols and fruit extracts of *L. ruthenicum* via PI3K/Akt pathway, providing potential treatment strategies for EIF in mice. Our results of KEGG enrichment analysis showed that PI3K-Akt signaling pathway, FoxO signaling pathway, and AMPK signaling pathway, were enriched with a total of 253, 106, and 91 target genes, respectively. Studies revealed the anti-fatigue effect of ginsenoside Rb1 on postoperative fatigue syndrome in elderly rats by its activation of PI3K/Akt pathway (Zhuang et al., 2014), while studies showed that the phosphoinositide 3-kinase (PI3K) played a vital role in skeletal muscle uptake signaling in skeletal muscles (Guo et al., 2019). In particular, the PI3K/Akt signaling pathway was closely related to various enzymatic activities in glucose metabolism to enhance glycolysis and to provide energy for the body during exercise (Xie et al., 2019). This was consistent with the findings revealed in our study of GO annotation. Furthermore, it was well-known that the PI3K-Akt signaling pathway was regulated by the activities of miRNAs in various biological processes (Jafari et al., 2019). These results were consistent with our findings revealing the regulatory functions of miRNAs in the PI3K-Akt pathway in response to EIF in mice treated with tea polyphenols and fruit extracts of *L. ruthenicum*. Moreover, the FoxO signaling pathway regulated by miRNAs is recognized as a potential treatment target for many diseases. For example, *let-7C* involved in PI3K/Akt/FoxO signaling pathway was identified as an anticancer gene for hepatocellular carcinoma (Li Y. et al., 2021). Our results of KEGG enrichment analysis revealed the regulatory functions of miRNAs involved in FoxO signaling pathway to alleviate the EIF in mice treated with tea polyphenols and fruit extracts of *L. ruthenicum*. Studies showed that the AMPK signaling pathway activated by the metabolic stress was probably involved in smooth muscle fatigue (Smith et al., 2017), while the AMPK signaling pathway was inhibited in mice with fatigue caused by chronic restraint stress-induced muscle atrophy (Wang et al., 2021). Furthermore, studies showed that the AMPK signaling pathway was involved in the improvement of muscle anti-fatigue ability in mice (Wen et al., 2021) and exercise-induced immunosuppression (Moir et al., 2008). Moreover, it was reported that the activation of the AMPK signaling pathway played the critical role in the recovery from exercise in patients with type 2 diabetes (Kjøbsted et al., 2016). It was reported recently that the AMPK signaling pathway played the central role in the regulation of mitochondrial dysfunction in cancer-related fatigue (Guo et al., 2021). Interestingly, studies showed that the extract of *L. ruthenicum* alleviated the nonalcoholic fatty liver disease by enhancing the AMPK signaling pathway (Lin et al., 2015), suggesting the significance of the AMPK signaling pathway in response to the treatment of mice with EIF by the fruit extracts of *L. ruthenicum*. Further studies are needed to verify the regulatory functions of these miRNAs and their target genes in these metabolic pathways in response to the treatments of tea polyphenols and fruit extracts of *L. ruthenicum* in mice with EIF, ultimately clarifying the molecular and pharmacological mechanisms regulating the therapeutic effects of tea polyphenols and fruit extracts of *L. ruthenicum* on the medical treatment of EIF in mice and providing scientific evidence to support the clinical application of both tea polyphenols and fruit extracts of *L. ruthenicum* in the treatment of EIF in professional athletes.

### 4.5 Effective components in the fruit extracts of *Lycium ruthenicum* and comparisons between the effects of tea polyphenols and fruit extracts of *L. ruthenicum* on the treatments of mice with EIF

Studies have shown that the fruit extracts of *L. ruthenicum* contain a large group of chemical components, e.g., flavonoids (e.g., anthocyanins), polysaccharides, phenolic acids, carotenoids, alkaloids, and essential oils (Girard et al., 2011; Wang H. et al., 2018). Due to their antioxidant activities, many of these chemical substances are identified as the functional components with therapeutical effects on various types of medical disorders (Zheng et al., 2011; Liu et al., 2013; Ni et al., 2013; Lin et al., 2015; Wang H. et al., 2018; Wang Y. et al., 2018; Zhang S. et al., 2019; Bowtell and Kelly, 2019; Cui et al., 2020; Deng et al., 2020; Lu et al., 2020; Luan et al., 2021; Luo et al., 2021; Tian et al., 2021). Therefore, it was naturally speculated that some of these chemical components, e.g., anthocyanins and polysaccharides, could be the effective components in the fruit extracts of *L. ruthenicum* in the alleviation of EIF. Studies have shown that the main components of tea polyphenols include catechins, flavonoids, phenolic acids, and flavonols, with catechins as the most abundant polyphenols, accounting for ∼65%–80% of the total tea polyphenols (Teng and Wu, 2017; Wang J. et al., 2018; Xing et al., 2019). Further studies are necessary to verify the findings revealed in our study and to provide evidence to support these speculations on the explicit functions of these potentially effective components (i.e., commonly found in both tea polyphenols and fruit extracts of *L. ruthenicum*) in the treatment of EIF.

Our results confirmed that both tea polyphenols and fruit extracts of *L. ruthenicum* showed the comparable levels of alleviation effects on the EIF (Figure 2). However, the molecular mechanisms underlying the therapeutic effects of both tea polyphenols and fruit extracts of *L. ruthenicum* are not known. It is naturally speculated that both tea polyphenols and fruit extracts of *L. ruthenicum* alleviate the EIF via different molecular mechanisms, simply due to the different chemical components in these two treatments and varied molecular patterns revealed by the miRNAs (Figure 3). Alternatively, it is still possible that both tea polyphenols and fruit extracts of *L. ruthenicum* could regulate their therapeutic effects on the alleviation of EIF via some shared molecular mechanisms (Figures 4–7). For example, our results showed that many shared metabolic pathways were identified by both GO annotation and KEGG enrichment analyses in the treatments of EIF of mice by both tea polyphenols and fruit extracts of *L. ruthenicum*. These results suggested that the alleviations of EIF in mice by both tea polyphenols and fruit extracts of *L. ruthenicum* were porbably regulated by some shared as well as varied molecular mechanisms. Further studies are necessary to identify the explicit molecular mechanisms underlying the therapeutic effects of both tea polyphenols and fruit extracts of *L. ruthenicum* on the alleviation of EIF.

Several limitations of this study were noted. First, due to the lack of a sedentary control group in the establishment of the mouse models of EIF, the results of biochemical factors in our study should be cautiously considered. This was because that the lack of comparative analysis between the model group and the control group could not rule out the possibility that the other factors, rather than the tea polyphenols and fruit extracts of *L. ruthenicum*, could also cause the same or similar effects on mice with EIF. Second, the differentially expressed microRNAs and their expression patterns identified in this study were not experimentally validated. These validations (e.g., quantitative real-time PCR analysis) are currently undergoing. With the experimental verification of these genetic components involved in the molecular mechanisms regulating the therapeutic effectiveness of fruit extracts of *L. ruthenicum* in the treatment of fatigue, it would significantly facilitate further exploration of the functions of these genetic components in developing clinical treatment targets of fatigue. Third, more biochemical factors (e.g., levels of lactic acid and blood urea nitrogen) related to fatigue could be explored to provide more evidence to support the findings revealed in our study. Fourth, the results of RNA-Seq analysis should be cautiously considered because the sample size of the RNA-Seq analysis was minimal in comparison with the same type of analysis, while increased sample size rather than the sequencing/read depth could definitely enhance the accurate identification of differentially expressed genes (Baccarella et al., 2018). Last, the explicit functions of the functional components in both tea polyphenols and fruit extracts of *L. ruthenicum* in the therapeutic treatment of EIF should be further investigated, ultimately identifying the main anti-fatigue substances, which could be further refined in both commercial and clinical applications in the treatment of fatigue.

## 5 Conclusion

In this study, the therapeutic effects of tea polyphenols and fruit extracts of *L. ruthenicum* on the treatment of mice with EIF were investigated. The results showed that both tea polyphenols and fruit extracts of *L. ruthenicum* significantly prolonged the swimming exhaustion time of mice and alleviated the EIF as evidently demonstrated by the changes in contents of a group of six fatigue-related biochemical indices (i.e., LDH, SOD, IL-1β, IL-2, IL-6, and TNF-α). These results revealed the therapeutic effects of tea polyphenols and fruit extracts of *L. ruthenicum* on the treatment of mice with EIF by preventing the oxidative stress and reducing inflammation. A group of 23 differentially expressed miRNAs (21 upregulated and 2 downregulated) involved in the molecular mechanisms regulating the EIF in mice were identified based on the high-throughput RNA sequencing technology in the experimental groups of mice treated with tea polyphenols and fruit extracts of *L. ruthenicum*. The regulatory functions of these miRNAs in the occurrence and treatment of EIF were further explored based on GO annotation and KEGG enrichment analyses to identify the metabolic pathways involved in the molecular response to the treatments of tea polyphenols of fruit extracts of *L. ruthenicum*. For the first time, the pathogenesis of EIF was explored based on the involvement of miRNAs with the group of miRNAs identified as the potential targets for the treatment of EIF in mice. These results provided strong scientific evidence to support the agricultural development of *L. ruthenicum* as well as the investigations and applications of tea polyphenols and fruit extracts of *L. ruthenicum* in the treatment of EIF in mice and eventually in humans. Further studies are needed to verify the regulatory functions of the miRNAs involved in the pathogenesis of EIF in mice and the pharmacological effects of tea polyphenols and fruit extracts of *L. ruthenicum* on the treatment of EIF in mice revealed in our study. Based on the advanced understanding of molecular mechanisms regulating the occurrence of EIF, it is expected that more clinically effective treatments could be explored and established to alleviate or ultimately prevent the EIF in humans, including the professional athletes.

## Data Availability

The datasets presented in this study can be found in online repositories. The names of the repository/repositories and accession number(s) can be found below: https://www.ncbi.nlm.nih.gov/, BioProject PRJNA858659: SAMN29723135 to SAMN29723142.
