# Supplementary material for: Molecular and biochemical investigations of the anti-fatigue effects of tea polyphenols and fruit extracts of Lycium ruthenicum Murr. on mice with exercise-induced fatigue
Source: Front Mol Biosci. 2023 Jun 20;10:1223411. doi: 10.3389/fmolb.2023.1223411 (PMC10319583; doi:10.3389/fmolb.2023.1223411)
Supplement: Supplementary file 1 [file DataSheet1.docx]

Supplementary Material

**Molecular and Biochemical Investigations of the Anti-Fatigue Effects of Tea Polyphenols and Fruit Extracts of *Lycium ruthenicum* Murr. on Mice with Exercise-Induced Fatigue**

**Yingxin Bi ^1, 2,^** ^†^**, Xianjun Liu^1,^** ^†^**, Yue Liu ^1, 2^, Mengyuan Wang ^1, 2^, Yaming Shan ^3, 4^, Yuhe Yin ^2^, Xianglong Meng ^5^, Fengjie Sun ^6,^ *, Hao Li ^1,^ *, Zhandong Li ^1,^ ***

^1^ College of Biological and Food Engineering, Jilin Engineering Normal University, Changchun 130052, China; 2201908056@stu.ccut.edu.cn (Y.B.); liuxianjun@jlenu.edu.cn (X.L.); 2202008073@stu.ccut.edu.cn (Y.L.); wangmengyuan11111@163.com (M.W.); [lihao@jlenu.edu.cn](mailto:lihao@jlenu.edu.cn) (H.L.); lizd591@jlenu.edu.cn (Z.L.)

^2^ School of Chemistry and Life Science, Changchun University of Technology, Changchun 130012, China; [yinyuhe@ccut.edu.cn](mailto:yinyuhe@ccut.edu.cn) (Y.Y.)

^3^ National Engineering Laboratory for AIDS Vaccine, School of Life Sciences, Jilin University, Changchun 130012, China; shanym@jlu.edu.cn (Y.S.)

^4^ Key Laboratory for Molecular Enzymology and Engineering, The Ministry of Education, School of Life Sciences, Jilin University, Changchun 130012, China

^5^ Department of Burns Surgery, The First Hospital of Jilin University, Changchun 130021, China; mengxljdyy@jlu.edu.cn (X.M.)

^6^ School of Science and Technology, Georgia Gwinnett College, Lawrenceville, GA 30043, USA; fsun@ggc.edu (F.S.)

^†^ These authors have contributed equally to this work and share first authorship.

*** Correspondence:**

Fengjie Sun: fsun@ggc.edu (F.S.);

Hao Li: lihao@jlenu.edu.cn (H.L.);

Zhandong Li: lizd591@jlenu.edu.cn (Z.L.)

# Supplementary Figures and Tables

## Supplementary Figures


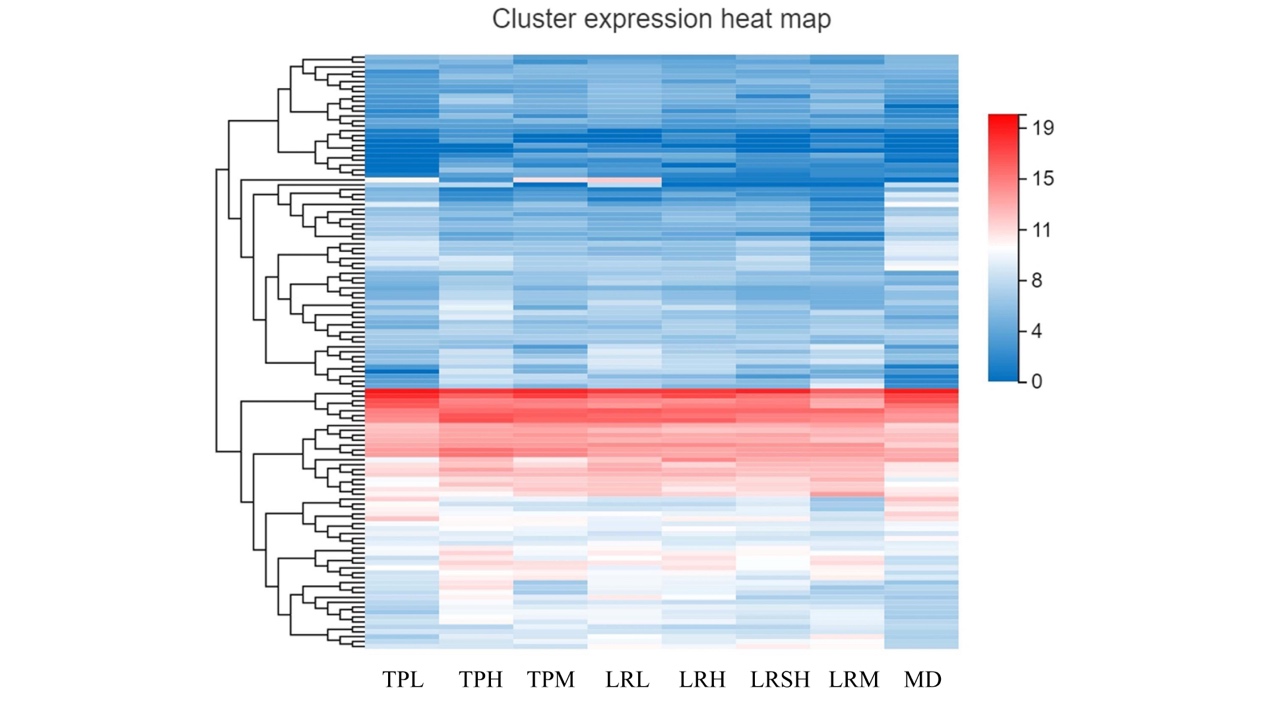


**Supplementary Figure 1.** Heatmaps of hierarchical clustering based on the microRNAs identified in three groups of mice treated with tea polyphenols of low-dose (TPL), medium-dose (TPM), and high-dose (TPH), respectively, and four groups of mice treated with fruit extracts of *Lycium ruthenicum* of low-dose (LRL), medium-dose (LRM), high-dose (LRH), and super high-dose (LRSH), respectively, and in the model control group (MD) fed with distilled water. Fold changes of gene expression are indicated by red bars showing up-regulation and blue bars showing down-regulation.

**
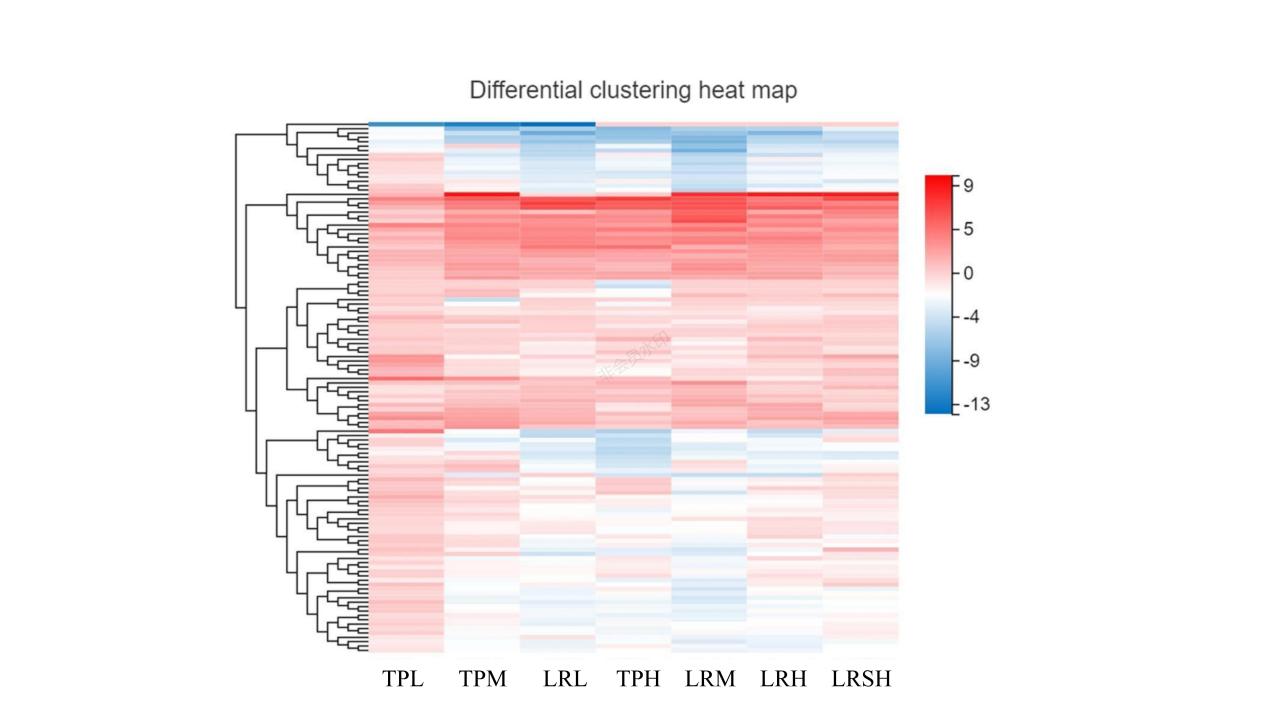
**

**Supplementary Figure 2.** Heatmaps of hierarchical clustering based on the differentially expressed microRNAs in three groups of mice treated with tea polyphenols of low-dose (TPL), medium-dose (TPM), and high-dose (TPH), respectively, and four groups of mice treated with fruit extracts of *Lycium ruthenicum* of low-dose (LRL), medium-dose (LRM), high-dose (LRH), and super high-dose (LRSH), respectively, in comparison with the model control group. Fold changes of gene expression are indicated by red bars showing up-regulation and blue bars showing down-regulation.


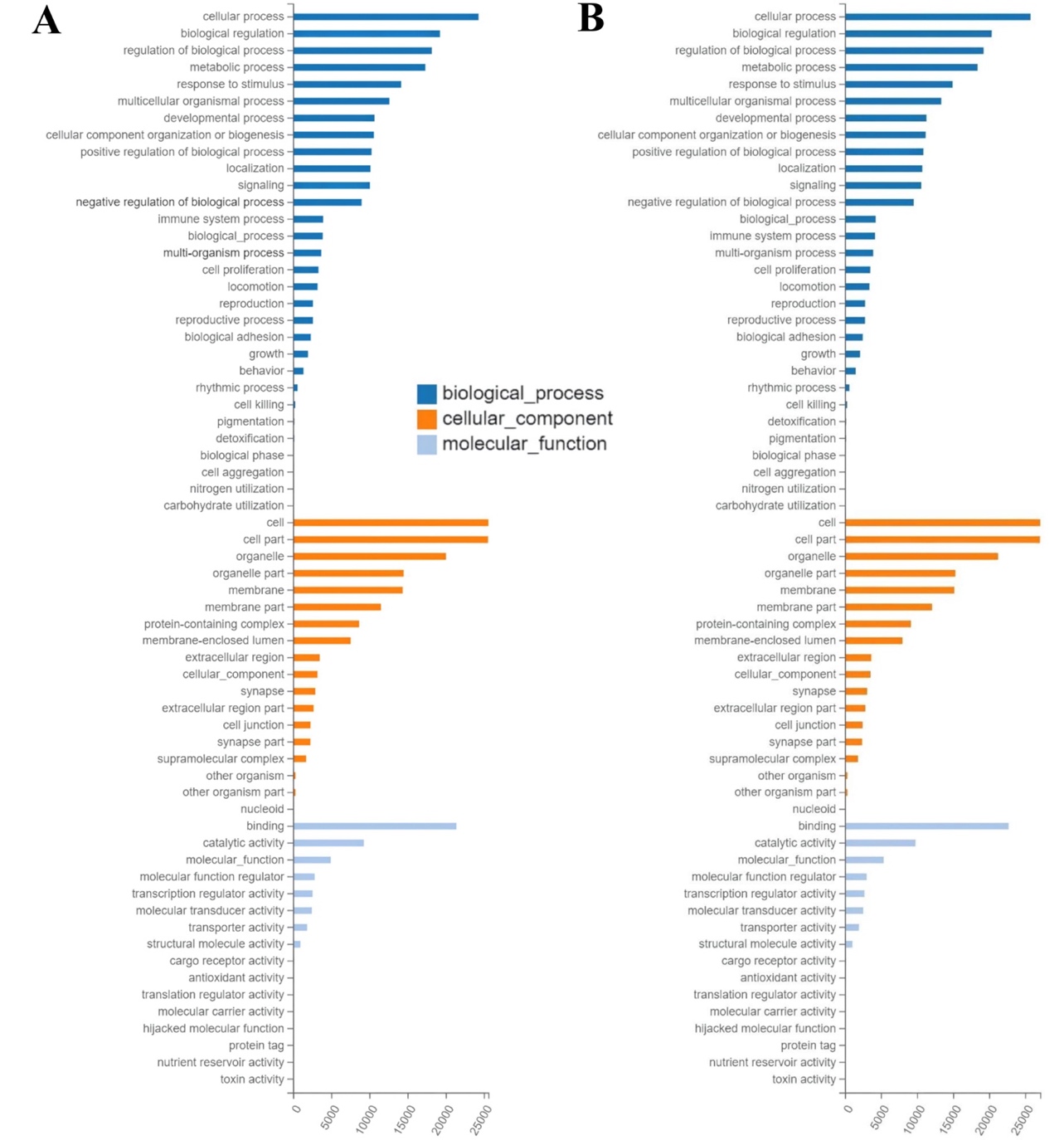


**Supplementary Figure 3.** Functional annotations based on the Gene Ontology (GO) database of the target genes of the differentially expressed microRNAs identified in two experimental groups of mice treated with tea polyphenols of medium-dose (**A**) and high-dose (**B**) in comparison with the model control group fed with distilled water, respectively, showing the number of differentially expressed microRNAs (X-axis) annotated in the three groups of GO terms (i.e., biological process, cellular component, and molecular function).


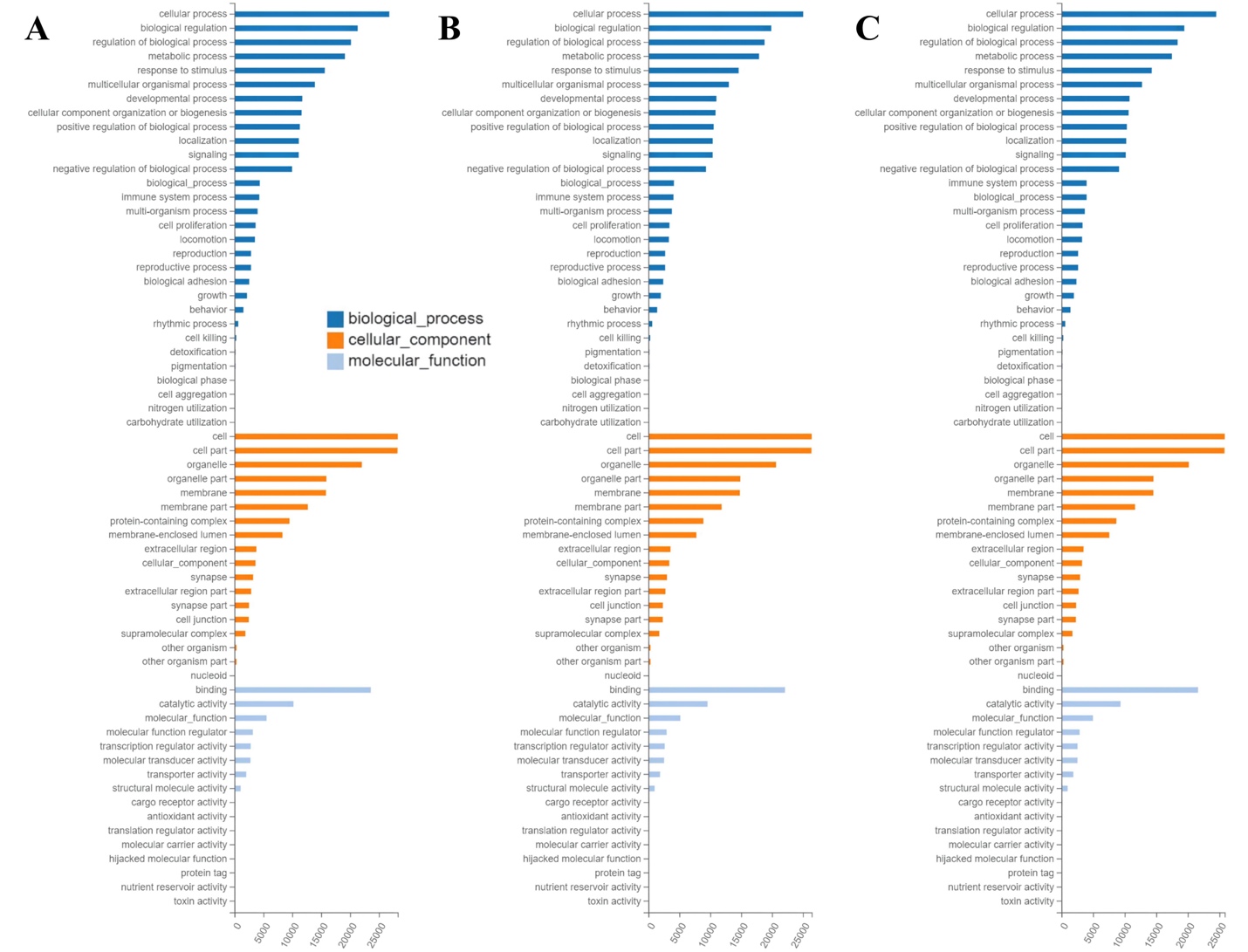


**Supplementary Figure 4.** Functional annotations based on the Gene Ontology (GO) database of the target genes of the differentially expressed microRNAs identified in three experimental groups of mice treated with fruit extracts of *Lycium ruthenicum* of medium-dose (**A**), high-dose (**B**), and super high-dose (**C**) in comparison with the model control group fed with distilled water, respectively, showing the number of differentially expressed microRNAs (X-axis) annotated in the three groups of GO terms (i.e., biological process, cellular component, and molecular function).

**
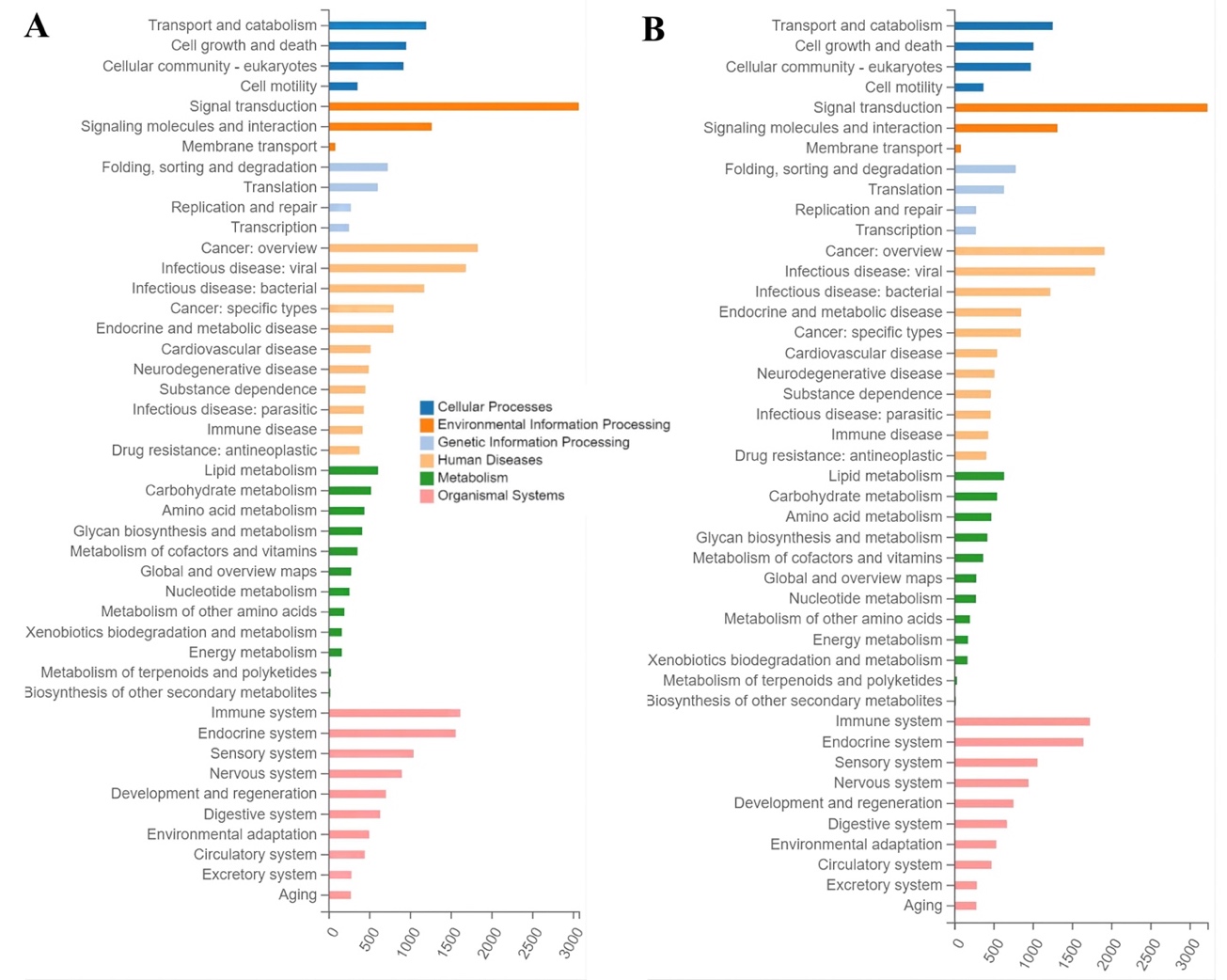
**

**Supplementary Figure 5.** Metabolic pathway enrichment analysis based on the Kyoto Encyclopedia of Genes and Genomes (KEGG) database of the target genes of the differentially expressed microRNAs identified in the two experimental groups of mice treated with tea polyphenols of medium-dose (**A**) and high-dose (**B**) in comparison with the model control group fed with distilled water, respectively, showing the number of differentially expressed microRNAs (X-axis) enriched in the six categories of metabolic pathways (i.e., cellular process, environment information processing, genetic information processing, human diseases, metabolism, and organismal systems) in the KEGG database.

**
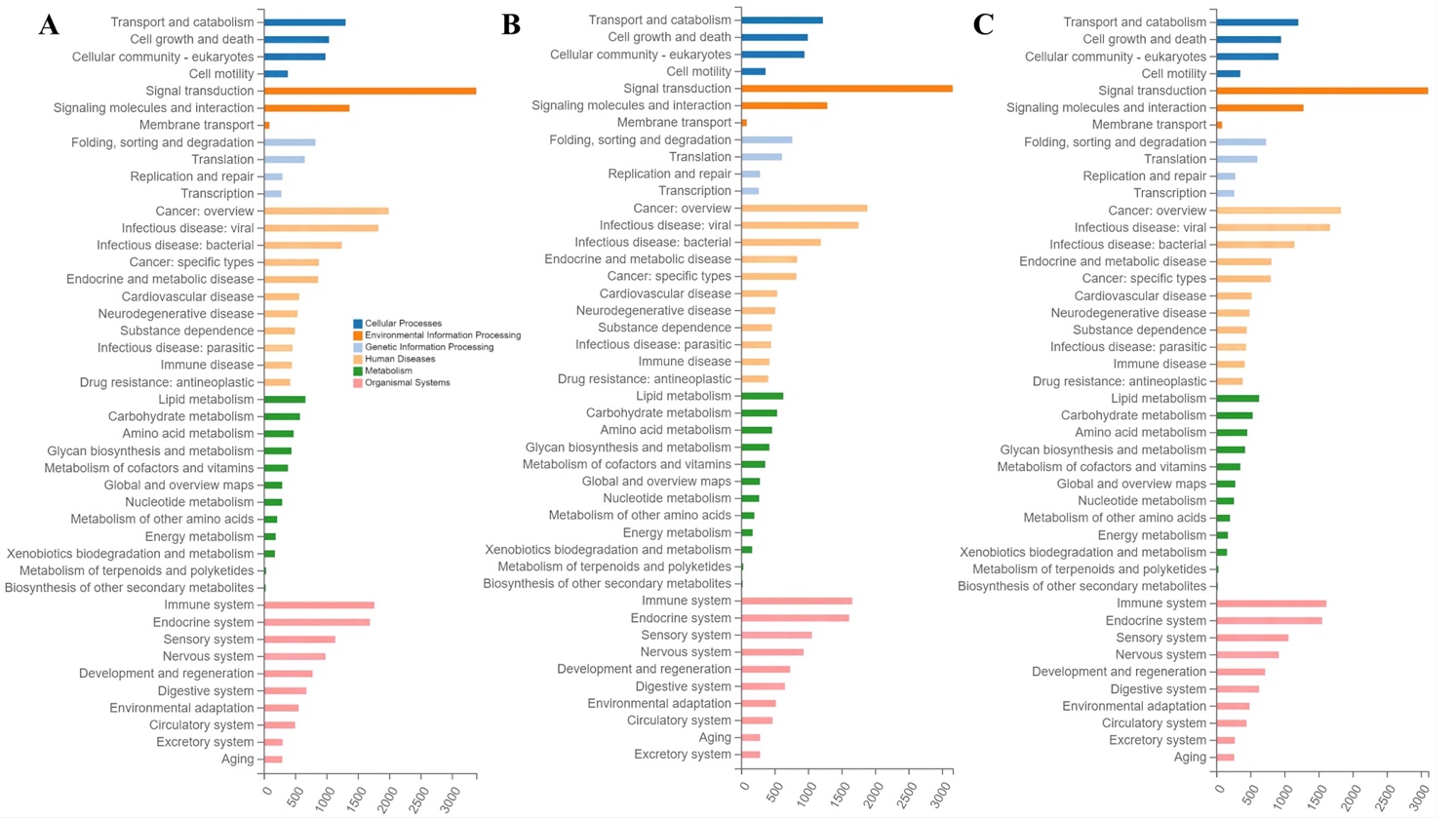
**

**Supplementary Figure 6.** Metabolic pathway enrichment analysis based on the Kyoto Encyclopedia of Genes and Genomes (KEGG) database of the target genes of the differentially expressed microRNAs identified in the three experimental groups of mice treated with extracts of *Lycium ruthenicum* fruits of medium-dose (**A**), high-dose (**B**), and super high-dose (**C**) in comparison with the model control group fed with distilled water, respectively, showing the number of differentially expressed microRNAs (X-axis) enriched in the six categories of metabolic pathways (i.e., cellular process, environment information processing, genetic information processing, human diseases, metabolism, and organismal systems) in the KEGG database.

## Supplementary Tables

**Supplementary Table 1.** Statistics of small RNAs sequenced in eight groups of mice treated with either tea polyphenols of low-dose, medium-dose, and high-dose or fruit extracts of *Lycium* *ruthenicim* of low-dose, medium-dose, high-dose, and super high-dose, respectively, based on the high-throughput RNA sequencing analysis.

| **Sample** | **Raw tag** | **Clean tag (%)** | **Q20 of Clean tag** | **Mapped tag** |
| --- | --- | --- | --- | --- |
| Model control | 25,165,824 | 21,507,174 (85.5) | 98.3% | 86.91% |
| Tea polyphenols |  |  |  |  |
| Low-dose | 25,165,824 | 22,196,457 (88.2) | 98.7% | 88.33% |
| Medium-dose | 25,165,824 | 23,457,064 (93.2) | 98.7% | 85.99% |
| High-dose | 25,165,824 | 22,736,123 (90.4) | 98.6% | 94.16% |
| *Lycium ruthenicum* |  |  |  |  |
| Low-dose | 25,165,824 | 23,803,216 (94.6) | 98.4% | 86.66% |
| Medium-dose | 25,165,824 | 23,489,217 (93.3) | 98.6% | 91.92% |
| High-dose | 25,165,824 | 22,609,048 (89.8) | 99.3% | 88.22% |
| Super high-dose | 25,165,824 | 23,541,403 (93.6) | 98.8% | 87.62% |

**Supplementary Table 2.** Expression patterns of differentially expressed microRNAs identified in mice with exercise-induced fatigue (EIF) treated with tea polyphenols and fruit extracts of *Lycium ruthenicum* based on GO annotation and expression analysis based on MiRanda and TargetScan. Symbols “↑” and“↓” indicate up-regulation and down-regulation, respectively.

| **microRNA** | **Target gene** | **GO term (GO ID)** |
| --- | --- | --- |
| miR-486a-3p ↓ | NM_001033324.3 | Skeletal system development (GO: 0001501) |
| miR-500-3p ↑ | NM_001111021.2 |  |
| miR-24-2-5p ↑ | NM_008780.2 |  |
| miR-24-2-5p ↑ | NM_009234.6 |  |
| miR-194-5p ↑ | NM_146144.4 |  |
| miR-19b-3p ↑ | NM_001014390.2 | Positive regulation of glycogen biosynthetic process (GO: 0045725) |
| let-7f-2-3p ↑ | NM_010570.4 |  |
| miR-144-3p ↑ | NM_001313894.1 | Positive regulation of glycogen catabolic process (GO: 0045819) |
| miR-144-3p ↑ | NM_010439.4 |  |
| miR-130a-3p ↑ | NM_010560.3 | Glycogen metabolic process (GO: 0005977) |
| let-7f-2-3p ↑ | NM_001081221.2 | Response to oxidative stress (GO: 0006979) |
| miR-500-3p ↑  miR-1a-1-5p ↑ | NM_001081549.2 |  |
| miR-499-5p ↑ | NM_001303408.1 |  |
| miR-141-3p ↑ | NM_007453.4 |  |
| miR-144-3p ↑ | NM_009730.3 |  |
| miR-144-3p ↑ | NM_011990.2 |  |
| miR-24-2-5p ↑ | NM_013820.3 | Negative regulation of reactive oxygen species metabolic process (GO: 2000378) |
| miR-142a-5p ↑ | NM_011580.4 | Positive regulation of reactive oxygen species metabolic process (GO: 2000379) |
| let-7f-2-3p ↑ | NM_010570.4 | Positive regulation of glucose metabolic process (GO: 0010907) |
| miR-500-3p ↑ | NM_001005767.4 | Regulation of reactive oxygen species metabolic process (GO: 2000377) |
